# Resolving Heterogeneity in the Diagnosis of Alzheimer’s Disease and its Progression Using Multimodal Data

**DOI:** 10.1007/s12031-026-02474-4

**Published:** 2026-02-04

**Authors:** Fuyan Hu, Nelson L. S. Tang, Haiying Wang, Huiru Zheng

**Affiliations:** 1 Department of Statistics, School of Mathematics and Statistics, Wuhan University of Technology, 122 Luoshi Road, Wuhan, 430070 China; 2 Department of Chemical Pathology and Li Ka Shing Institute of Health Sciences, Faculty of Medicine, The Chinese University of Hong Kong, Hong Kong SAR, 999077 China; 3 Functional Genomics and Biostatistical Computing Laboratory, CUHK Shenzhen Research Institute, Shenzhen, 518000 China; 4 Hong Kong Branch of CAS Center for Excellence in Animal Evolution and Genetics, School of Biomedical Sciences, The Chinese University of Hong Kong, Hong Kong SAR, 999077 China; 5 School of Computing, University of Ulster, Belfast, BT15 1ED United Kingdom

**Keywords:** Alzheimer's disease, Biomarkers, Similarity network fusion, Spectral clustering, Subtypes

## Abstract

Alzheimer’s disease (AD) is a complex and diverse illness that makes early detection extremely difficult. Most existing research utilizes data to identify biomarkers and more homogeneous subgroups to improve the detection, prediction of progression, and prognosis of AD. However, AD still suffers from a lack of appropriate biomarkers for early symptom detection and blurred boundaries between different subgroups. Here, an unsupervised clustering method known as similarity network fusion (SNF) was employed to analyze multimodal data from 972 subjects, including 370 with cognitively normal (CN), 565 with mild cognitive impairment (MCI), and 37 patients with AD. First, we constructed a similarity network for subjects using cognitive scores, genetics, and magnetic resonance imaging (MRI) related data, respectively. Then the SNF fusion method was employed to integrate the data, and spectral clustering was used to find subgroups sharing similarities across modalities. Our results indicated that the approach accurately diagnosed both current and prospective AD (~ 90%). Notably, we successfully identified two MCI subtypes with biological and clinical significance, validated by longitudinal studies of cognitive, clinical, fluid biomarkers and MRI-related features, dementia diagnosis, and pseudo-trajectory analysis. We also observed many dysregulated processes and signaling pathways between MCI subtypes, such as the GnRH signaling pathway, VEGF signaling pathway, and insulin signaling pathway. Overall, our research offers a distinctive viewpoint on the diversity of AD, and the more specific subtypes of MCI help create customized treatment plans.

**Supplementary Information:**

The online version contains supplementary material available at 10.1007/s12031-026-02474-4.

## Introduction

Dementia is characterized by progressive decline in memory and cognition, with Alzheimer’s disease (AD) being the most common type. According to estimates, the number of people with dementia will rise from 50 million in 2019 to 150 million by 2050 (Li et al. 2024; Brookmeyer et al. 2007). Dementia-related deaths worldwide rose from 0.56 million in 1990 to 1.62 million in 2019, with a total of 4.91 million expected by 2050 (Li et al. 2024). In addition, the number of deaths due to dementia under the age of 70 is expected to rise to 0.18 million by 2050 (Li et al. 2024). Although available medications (Wong et al. 2023; Szekely et al. 2004) and non-pharmacologic prevention (Bhatti et al. 2019) can temporarily reduce the exacerbation of dementia and the incidence of AD to some extent, there is no cure for AD due to its complexity.

The typical pathological feature of AD is the abnormal accumulation of Amyloid-beta (Abeta) plaques and tau protein tangles in the brain (Hardy and Higgins 1992; Medeiros et al. 2011), both of which can be reflected by amyloid and tau loads on PET and cerebrospinal fluid tests. Previous research has focused on identifying biomarkers related to AD. Several risk factors, such as the apolipoprotein E4 (APOE4) gene (Reinvang et al. 2013), age (Guerreiro and Bras 2015), family history (Cupples et al. 2004; Tabuas-Pereira et al. 2024), lifestyle (Weih et al. 2007; Flicker 2010), cognitive problems, and brain atrophy (Killiany et al. 2000), have been identified using genetic data, behavioral data, cognitive performance measurements and neuroimaging. However, existing studies have shown that AD is heterogeneous, and its causes are complex, and it cannot be explained by a single biomarker alone (Komarova and Thalhauser 2011; Dubois et al. 2023).

Artificial intelligence technology provides many valuable insights in early diagnosis, personalized treatment, and prognostic assessment of AD through the extensive application of medical data (Kale et al. 2024; Zhang et al. 2023). For example, support vector machine (SVM) classifier was used to classify CN/AD, and the accuracy was 85.71% (Arya et al. 2023). In deep learning, the accuracy can reach 98.6% using the convolutional neural network (CNN) classifier and 91.2% using the recurrent Neural Network (RNN) classifier (Arya et al. 2023). A systematic review and meta-analysis demonstrates that CNN algorithms applied to structural MRI achieve high diagnostic accuracy in differentiating AD from normal cognition and moderate accuracy in distinguishing MCI from normal cognition, though performance is lower for predicting progression from MCI to AD, highlighting both the potential and current limitations of CNN-based radiomics for AD diagnostics (Dong et al. 2025). A study introduces Subtype and Stage Inference (SuStaIn), a machine learning method that simultaneously identifies disease subtypes and their temporal progression patterns using cross-sectional regional brain volumes from MRI data (Young et al. 2018). While more machine learning and deep learning technologies are being utilized to investigate AD, we must continue to focus on balancing model explainability and complexity.

Currently, methods for integrating and analyzing multimodal data have been applied to the study of AD with remarkable results. By intelligently combining genetic information and brain scans, Brand et al. proposed an innovative joint multimodal longitudinal regression and classification method to identify cognitive states and the underlying biological mechanisms in AD (Brand et al. 2020). Ning et al. presented a relationship-induced multimodal shared representation learning method designed to fuse MRI and positron emission tomography (PET) data for more accurate identification of AD (Ning et al. 2021). Jiao et al. proposed a multimodal data fusion framework combining deep auto-encoder and self-representation for early diagnosis of AD (Jiao et al. 2024). Avelar-Pereira et al. constructed a multilayer network integrating structural MRI, PET, cerebrospinal fluid, cognition, and genetics data, and then successfully identified AD and its subtypes by applying a multilayer community detection algorithm, providing a unique perspective on the heterogeneity of AD (Avelar-Pereira et al. 2023). Although integration algorithms for multimodal data provide a new approach to understand AD, effective implementation of learning complementarity and information fusion in multimodal data remain challenges due to the limited number of samples, excessive feature dimensionality, and heterogeneity among different data types.

In this study, we used the similarity network fusion (SNF) (Wang et al. 2014), an unsupervised clustering method, to integrate data from multiple modalities into an aggregated network that can effectively capture the heterogeneity of AD. The advantages of this technique have been validated in numerous studies (Yang et al. 2023; Li et al. 2021). In our work, SNF was applied to three modality data from 972 subjects from the Alzheimer’s Disease Neuroimaging Initiative (ADNI) to detect AD and subtypes, including cognitive, genetic, and MRI information. We then characterized these subtypes by correlating them with factors such as neuropathology, cognitive performance, and longitudinal data. Furthermore, we also identified the differences in biological processes and signaling pathways between subtypes by gene set variation analysis (GSVA). In addition, we performed pseudo-temporal construction and trajectory analysis with clinical data to validate changes in severity across subtypes.

## Materials and methods

### Study Samples

Data used in this work were obtained from the Alzheimer’s Disease Neuroimaging Initiative (ADNI) database (https://adni.loni.usc.edu/). ADNI is a multicenter study launched in 2003 to detect and validate biomarkers for Alzheimer’ s disease (AD) research. The ADNI database comprises various data types, such as clinical data, cognitive tests, genetics data, demographics, neuroimaging data, and biofluid biomarkers. Our study was based on 972 subjects with at least four years of follow-up information. At baseline, 37 of these individuals were diagnosed with AD, 565 with MCI, and 370 with cognitively normal (CN).

### Multimodal Data Information and Preprocessing

Cognitive data included the Alzheimer’s Disease Assessment Scale (ADAS), Rey’s Auditory Verbal Learning Test (RAVLT), the modified preclinical Alzheimer cognitive composite using digit symbol substitution test (mPACCdigit), the modified preclinical Alzheimer cognitive composite using trail-making test part B (mPACCtrailsB), Logical Memory-Delayed Recall Total Number of Story Units Recalled (LDELTOTAL), Trail Making Test Part B Time (TRABSCOR), and Functional Assessment Questionnaire (FAQ). As the Clinical Dementia Rating Scale (CDRSB) and the Modified Mental State Examination (MMSE) are commonly used for diagnostic purposes, we did not use them for community screening in order to avoid bias and high specificity. We presented the between-group differences in these cognitive scores to validate and explain the communities. In terms of genetic information, participants’ APOE gene information and their polygenic hazard scores (PHS) were covered. As for MRI-related information, seven variables were retained, including the volume of the Ventricles, Hippocampus, Whole Brain, Entorhinal, Fusiform gyrus, Middle temporal gyrus (MidTemp), and intracerebral volume (ICV). Imputation for variables with a missing rate of 20% or less was done using the MissForest method (Stekhoven and Buhlmann 2012). The details of these variables for the 972 samples are outlined in Table S1.

### Community Detection with SNF

The SNF method was used to integrate multimodal data by the ‘SNFtool’ R package (v2.3.1) with recommended parameters *K* = 40, alpha = 0.5, and *T* = 10 (where *K* is the number of neighbors; alpha is a hyper-parameter; *T* is the total number of algorithmic iterations) (Wang et al. 2014). SNF first built sample similarity matrices for each data modality independently and then updated and integrated these matrices in a nonlinear combination to generate a fused similarity network. The fused similarity network was then utilized to divide study participants into subgroups using spectral clustering (Park and Zhao 2018). Prior to constructing the similarity matrices for each data modality, we normalized the data for each modality using the ‘standardNormalization’ function from the SNFtool package, which scales each column to have a mean of 0 and a standard deviation of 1. The rotation cost and eigen-gap approaches were used to determine the optimal cluster size (2 to 8 clusters) (Park and Zhao 2018; Huang et al. 2013).

### Robustness of Clustering

The robustness of clustering was tested by using a leave-one-out strategy. First, we ran SNF on the entire cohort of study participants and then grouped patients into two subgroups using spectral clustering. Next, one patient at a time was deleted from the data to construct a new fused similarity network by the SNF method. Then, patients were divided into two subgroups by spectral clustering. Each new patient group was compared to the one from the entire cohort. The accuracy and normalized mutual information (NMI) (and 2009) were calculated for the comparison groupings. NMI is calculated using the function calNMI() from the ‘SNFtool’ R package (v2.3.1).

### Survival Analysis

To compare the time required to transition from MCI to AD in patients with different MCI subtypes, survival analysis was performed on MCI subtypes to quantify the effectiveness of clustering further. Because the exact time at which patients with MCI progress to patients with AD is unknown, the follow-up time at which AD was first diagnosed was used. For MCI patients who did not develop AD, the final follow-up time was chosen. Kaplan-Meier analysis was performed on the time to development of AD in patients with different subtypes of MCI.

### Clinical Trajectory Analysis for Patient Subgroups

ClinTrajan, a powerful tool for clinical trajectory analysis based on elastic master charts (Golovenkin et al. 2020), was performed on all study subjects to determine and evaluate the progression trajectory of AD. ‘ClinTrajan’ python package was used to quantify the pseudotime of dementia progression states based on cognitive data, genetics data, and MRI-related information. The parameters for principal tree calculation were set as: the number of nodes = 50, α = 0.01, µ = 0.1, λ = 0.05. A trajectory was selected when it starts with CN (lowest risk of dementia) and ends with AD patients.

### Gene Set Variation Analysis (GSVA)

The blood gene expression data from ADNI were employed to perform GSVA. For transcripts with multiple probes, their expression levels were represented by​ the average of the corresponding probes. Using log-transformed gene expression data, we performed gene set enrichment analysis with the R package GSVA (v1.48.3) on hallmark KEGG (Kyoto Encyclopedia of Genes and Genomes) pathways and GO (Gene Ontology) terms from the MSigDB (https://www.gsea-msigdb.org/gsea/msigdb). The R package Limma (v.3.56.2) was used to compare the differences between different subtypes.

### Statistical Analyses

Chi-square tests were utilized to assess differences in categorical variables. Wilcoxon signed-rank tests were applied to evaluate differences between communities in the variables of interest. These included not only all the interested cognitive tests such as CDRSB, ADAS11, ADAS13, ADASQ4, and MMSE scores, but also the subject’s brain volume in the hippocampus, whole brain, and so on. We also compared CSF β-amyloid (ABETA), phosphorylated tau (PTAU), total tau (TAU), and PET imaging results, such as florbetapir (AV45) and the average FDG-PET of angular, temporal, and posterior cingulate (FDG).

## Results

### The SNF-based Method Identified Healthy and AD Cases Accurately

**Fig. 1:**
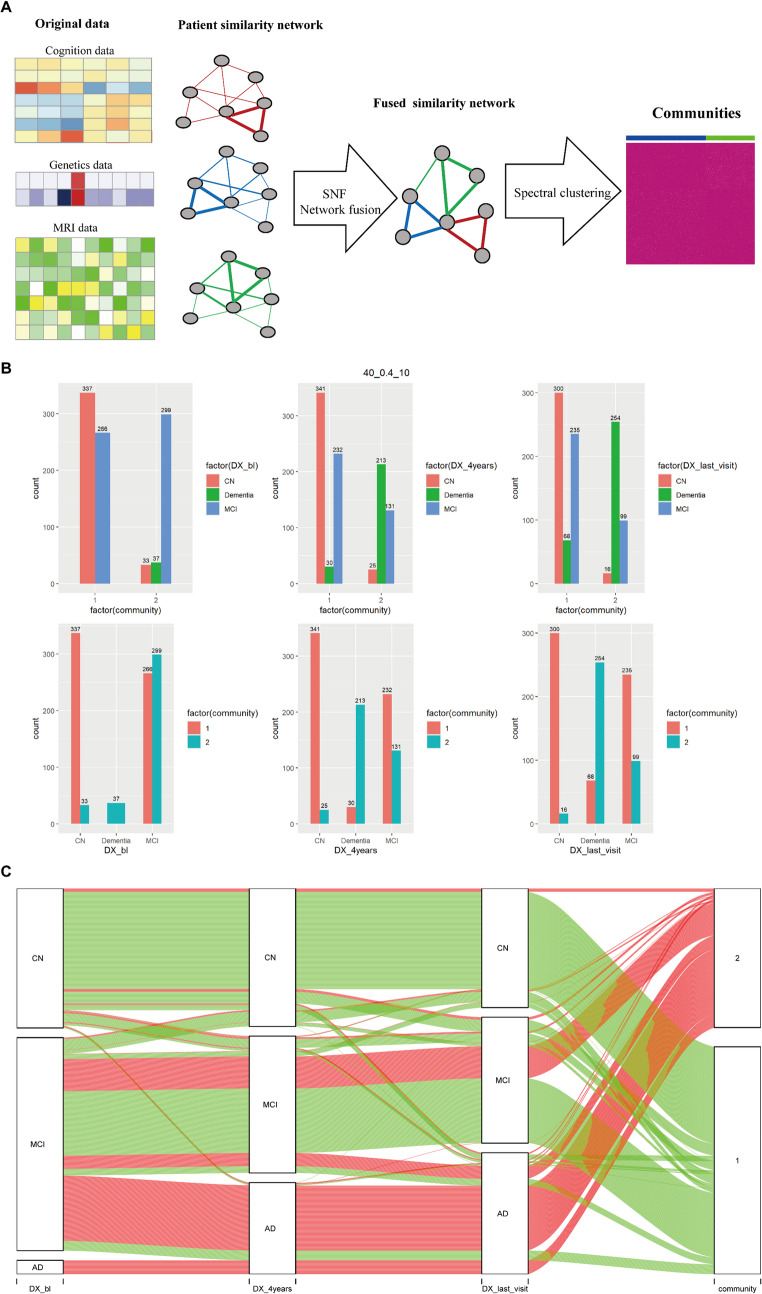
Overview of the SNF-based method used in the study and the resulting communities. (**A**) The framework of SNF method. (**B**) The distribution of communities for each diagnosis group. The first column displays the distribution of communities based on the diagnosis of baseline, while the second column shows the results based on the diagnosis at the fourth year of follow-up. In the third column, the distribution of communities using last visit diagnostics is shown. (**C**) Alluvial plot showing the overlap of communities and the diagnosis based on different time points

Spectral clustering was performed on the SNF-integrated patient similarity matrix, resulting in two communities (Fig. 1 and Table S2), with one (C1) including the majority (91.1%) of CN individuals and the other (C2) including all AD cases diagnosed at baseline. Since individuals were followed longitudinally, the results were also checked at fourth year of follow-up and the last visit: (1) According to the diagnosis at the fourth year of follow-up, C1 covered the majority (93.2%) of CN persons, whereas C2 accounted for 87.7% of all AD patients; (2) According to the diagnosis at the last visit, C1 accounted for the majority (94.9%) of CN persons, whereas C2 contained 78.9% of all AD patients. These results demonstrated that the SNF-based community detection method allows for accurate identification of present (100%) and future AD (≈ 88% at the fourth year of follow-up). The alluvial plot (Fig. 1C) showed that the number of subjects with CN and MCI decreased over time, while the number of subjects with AD increased.

Differences between communities were examined on all the 28 variables of interest, considering comparisons to be significant if they passed the Bonferroni correction ($p=0.00179$). For baseline data, differences in APOE status, demographic characteristics, and cognitive scores were compared between the C1 and C2 subgroups. C1 and C2 showed significant differences in APOE status (${\chi ^2}=~216.64$, $p$=9.07$\times$10^-48^), with fewer subjects carrying one or two alleles in C1. C1 and C2 had differences in age ($t=~ - 3.223$, $p=1.32 \times {10^{ - 3}}$) and education ($t=~3.853$, $p$=1.27$\times$10^-4^), with C2 had older subjects and lower education. The communities also displayed significant differences in cognitive scores, such as CDRSB (${\chi ^2}=~262.73$, $p$=1.44$\times$10^-48^), MMSE ($t=16.785$, $p$=8.92$\times$10^-51^), ADAS13 ($t=~ - 24.542$, $p$=2.25$\times$10^-91^), and RAVLT_forgetting ($t=~ - 8.788$, $p$=7.80$\times$10^-18^), with C2 performing worse than C1. In addition, the results revealed that C2 had higher levels of atrophy in the hippocampus ($t=19.979$, $p$=1.73$\times$10^-70^), entorhinal cortex ($t=18.088$, $p$=7.57$\times$10^-60^), fusiform ($t=8.079$, $p$=2.50$\times$10^-15^), MidTemp ($t=9.944$, $p$=6.50$\times$10^-22^) and whole brain ($t=4.340$, $p$=1.62$\times$10^-5^) compared to C1. Moreover, C2 also had higher level of ventricular enlargement ($t= - 7.629$, $p$=8.50$\times$10^-14^). Additionally, C2 had higher levels of tau ($t= - 9.244$, $p$=4.01$\times$10^-19^) and pTau ($t= - 10.181$, $p$=1.66$\times$10^-22^) in their CSF, and lower levels of amyloid-β ($t=17.012$, $p$=1.39$\times$10^-56^). Finally, those in C2 showed significantly higher AV45 ($t= - 15.621$, $p=5.84 \times {10^{ - 48}}$) and lower FDG PET ($t=13.175$, $p$=1.21$\times$10^-35^) uptake.

### Robustness Analysis of Patient Clustering

The leave-one-out robustness test, which removed each patient sequentially, had an average accuracy of 99.8% (SD = 0.1%) and an average normalized mutual information (NMI) of 98.1% (SD = 0.95%). That is, when we removed a patient from the dataset, the remaining patients often belonged to the same cluster as the results of using the entire patient cohort. The clustering results after removing patients one by one were consistent, indicating that the proposed pipeline was robust to sample variations.

### Longitudinal Analysis of Different Communities Moving to AD

We then examined community differences in AD progression. Overall, the mean follow-up time was 8.1 ± 3.7 years for CN participants, 6.3 ± 3.1 years for MCI patients, and 4.6 ± 2.0 years for AD patients. The two communities displayed different patterns in progression to AD (Fig. S1A and Fig. S2). In C1, 50.25% individuals were stable CN, meaning they were CN at baseline and were still diagnosed with CN four years later, while only 5.42% of C2 were stable CN. The conversion of MCI to AD was only 4.31% in C1, while in C2 this number reached 46.89% (Fig. S2A). Of the 37 ADs in C2, they were still AD four years later. Four years later, for all subject with CN in C1, 89.91% of them were still CN, and 8.9% became MCI, and 1.19% became AD; for all subject with CN in C2, the proportion for the three disease states (i.e., CN stable, CN to MCI, and CN to AD) were 60.61%, 30.3%, and 9.09%, respectively (Fig. S2B). As for all patients with MCI in C1, the percentages for the three disease states (i.e., MCI stable, MCI to CN and MCI to AD) were 75.94%, 14.29%, and 9.77%, respectively; for all patients with MCI in C2, the percentages for the three disease states were 40.47%, 1.67%, and 57.86%, respectively (Fig. S2C). Four years later, of all MCI participants who developed AD (*n* = 199), 13.07% (*n* = 26) belonged to C1 and 86.93% (*n* = 173) belonged to C2 (Fig. S1B). Overall, 10% of C1 (*n* = 60) changed to a more severe disease state (including three conditions: CN to MCI, CN to AD, and MCI to AD) four years later, while in C2 this number was as high as 50.4% (*n* = 186). According to the diagnosis at the last visit, 20.4% of C1 (*n* = 123) and 62.1% of C2 (*n* = 229) turned into a more severe disease state.

### Comparing Subjects with CN in Different Communities

**Fig. 2:**
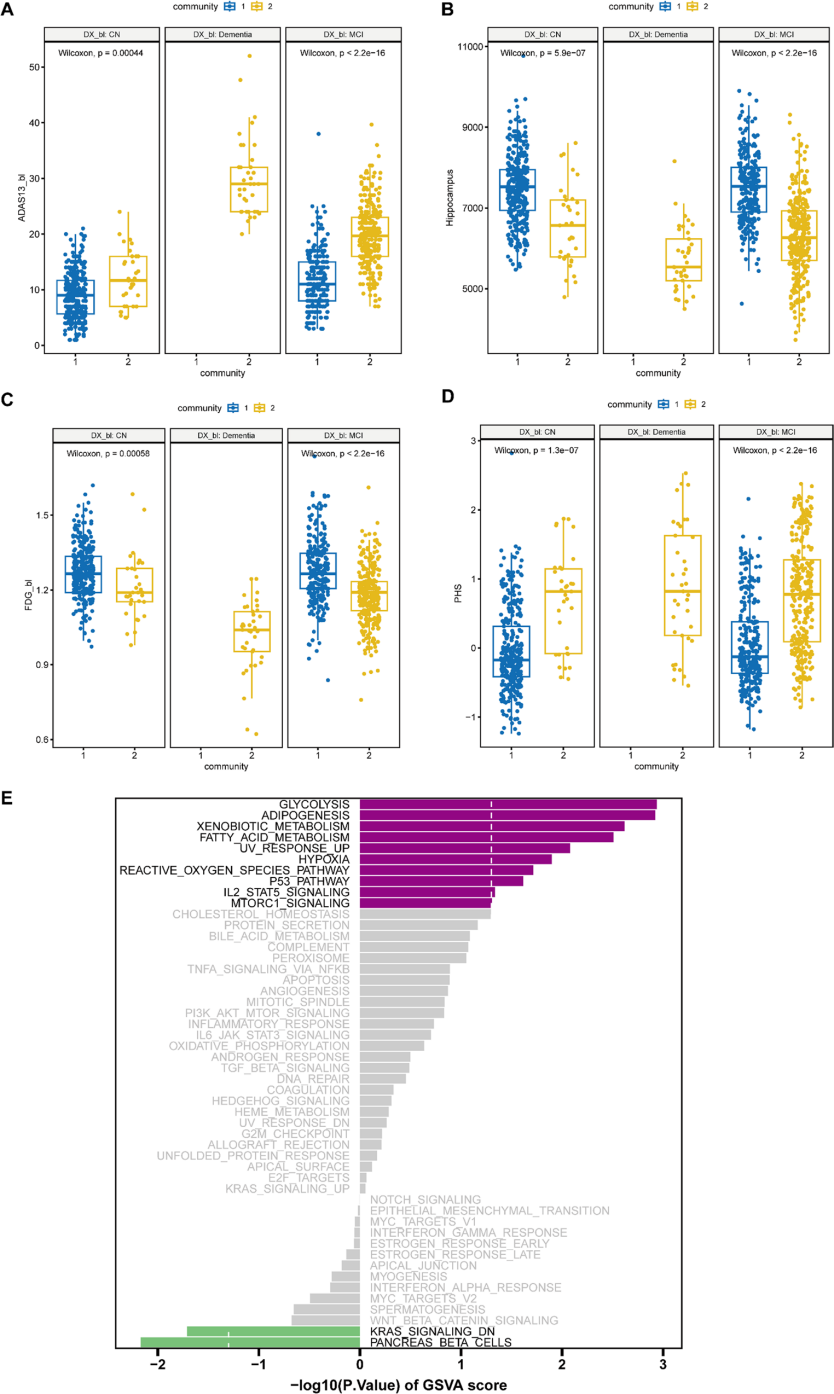
Comparing the differences between C1 and C2 by diagnosis group. (**A**) ADAS13 score; (**B**) Hippocampus; (**C**) FDG; (D) PHS; (**E**) GSVA demonstrates pathway differences between CN-C2 and CN-C1. CN, cognitively normal

The differences between subjects with CN in different communities (referred to as CN-C1 and CN-C2) were examined. There were 33 CN subjects (8.9%) in C2 and 337 (90.1%) in C1. Interestingly, the results of Wilcoxon signed-rank tests suggested that the subjects with CN in C2 had higher scores in ADAS13 score, TRABSCOR score, and mPACCtrailsB, higher level of atrophy in the hippocampus, entorhinal cortex, and higher level of ventricular enlargement, and lower FDG PET uptake, and higher polygenic hazard score (PHS) (Fig. 2A-D, Fig. S3-S5).

To get a more comprehensive comparison between CN-C1 and CN-C2, the hallmark gene sets from the Molecular Signatures Database (MsigDB) were selected to calculate the enrichment scores of each pathway through GSVA (Gene Set Variation Analysis). GSVA results showed that CN-C2 was significantly up-regulated in many important pathways, such as glycolysis, adipogenesis, p53 and mTORC1 signaling, and CN-C2 was significantly down-regulated in pancreatic β-cell and KRAS_SIGNALING_DN (Fig. 2E).

### Comparing MCIs in Different Communities Based on Baseline Data

In the case of MCI, there were 266 patients with MCI in C1 and 299 patients with MCI in C2 at baseline (referred to as MCI-C1 and MCI-C2) (Fig. 1B). We first compared baseline cognitive scores, MRI information, and CSF concentrations in the MCI-C1 and MCI-C2 subgroups (Fig. 2A-D, Fig. S3-S5). The two subgroups had significant differences on each of the 14 cognitive scores. Specifically, MCI-C2 had higher score in CDRSB, ADAS11, ADAS13, ADASQ4, RAVLT_perc_forgetting, RAVLT_perc_forgetting, TRABSCOR, and FAQ, while MCI-C2 had lower score in MMSE, RAVLT_immediate, RAVLT_learning, LDELTOTAL, mPACCdigit, and mPACCtrailsB. Furthermore, compared with MCI-C1, the hippocampus, entorhinal cortex, fusiform gyrus, MidTemp and whole brain atrophy were more severe in MCI-C2 group. And MCI-C2 also showed a larger degree of ventricular enlargement. In addition, MCI-C2 had higher levels of tau and pTau in their CSF, and lower levels of amyloid-β. Finally, AV45 was significantly increased and FDG-PET uptake was significantly decreased in MCI-C2.

**Fig. 3:**
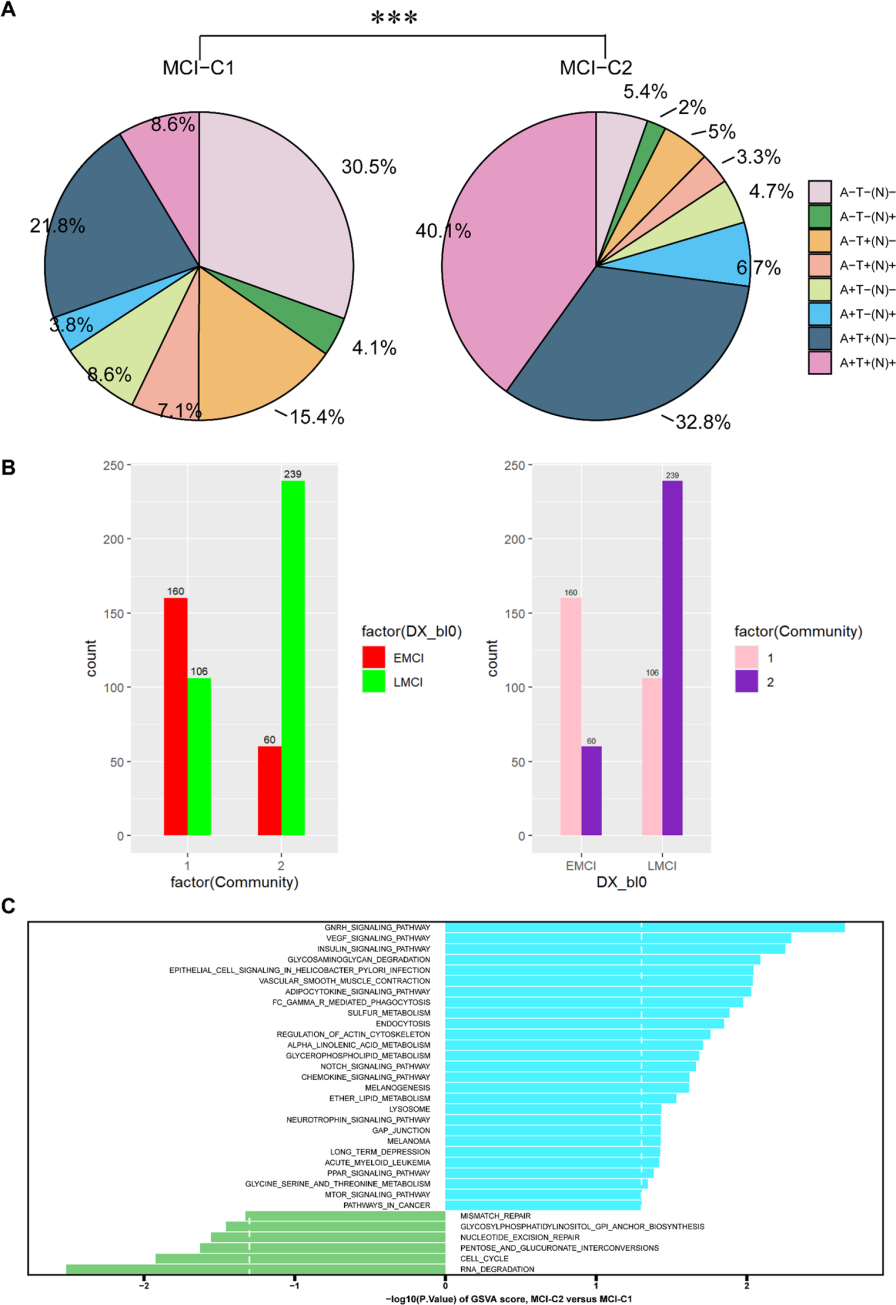
Validation of the MCI subgroups. (**A**) Participants in the MCI-C1 and MCI-C2 groups were classified as positive or negative for ABETA (A), p-Tau (T) and FDG-PET (N) biomarkers. The pie charts show the biomarker combinations in the MCI-C1 and MCI-C2 subgroups. The distributions differed significantly between the two subgroups. ***p < 0.001. (**B**) Bar charts illustrate the overlap between our identified MCI subtypes and early and late MCI (EMCI and LMCI) subtypes. (**C**) GSVA demonstrates pathway differences between MCI-C2 and MCI -C1

We then investigated each subgroup’s prevalence of different biomarker profiles based on three biomarker groups, which stand for the three distinct pathologic processes associated with AD that are measurable by three biomarkers: ABETA (A), pathologic tau (T), and neurodegeneration/neuronal damage (N). In this study, CSF ABETA was used as the biomarker of ‘A’; CSF pTau was used as the biomarker of ‘T’ and FDG-PET was used as the biomarker of ‘(N)’. Then, eight potential biomarker profiles were generated by binarizing each of the three biomarker groups into normal/abnormal (+/−) based on previously published cutoff values (Jack et al. 2018; Kwak et al. 2021; Ou et al. 2019), such as A + T−(N) − and A + T+(N)−. The prevalence of biomarker profiles was significantly different between MCI-C1 and MCI-C2 (${\chi ^2}=140.02$,$p<2.2 \times {10^{ - 16}}$) (Fig. 3A, Table S3). In particular, the type of A + T+(N) + was more common in MCI-C2 (40.1%) than in MCI-C1 group (8.6%). In contrast, the type of A-T-(N)- was more prominent in the MCI-C1 (30.5%) than in the MCI-C2 group (5.4%). Subjects with abnormal CSF ABETA or pTau or FDG-PET were more common in MCI-C2 (CSF ABETA: 84.2%; pTau: 81.3%; FDG-PET: 53.3%) than in the MCI-C1 group (CSF ABETA: 42.8%; pTau: 53.0%; FDG-PET: 23.7%). We also compared our MCI-C1 and MCI-C2 with the early and late MCI subtypes (EMCI and LMCI) (Fig. 3B). Of the 299 MCI-C2 cases, 239 (79.9%) were LMCI, whereas only 106 (39.8%) of the 266 MCI-C1 cases were LMCI.

GSVA results showed that MCI-C2 was significantly up-regulated in many important KEGG pathways, such as GnRH signaling pathway, VEGF signaling pathway, insulin signaling pathway and Glycosaminoglycan degradation, while it was significantly down-regulated in Glycosylphosphatidylinositol (GPI) anchor biosynthesis and the pentose and glucuronate interconversion pathways, compared with MCI-C1 (Fig. 3C). MCI-C2 was significantly up-regulated in many important GO BP terms, such as positive regulation of vascular associated smooth muscle cell differentiation and regulation of sphingolipid biosynthetic process (Fig. S6A).

### Longitudinal Analysis of the MCI Subgroups in Different Features

**Fig. 4:**
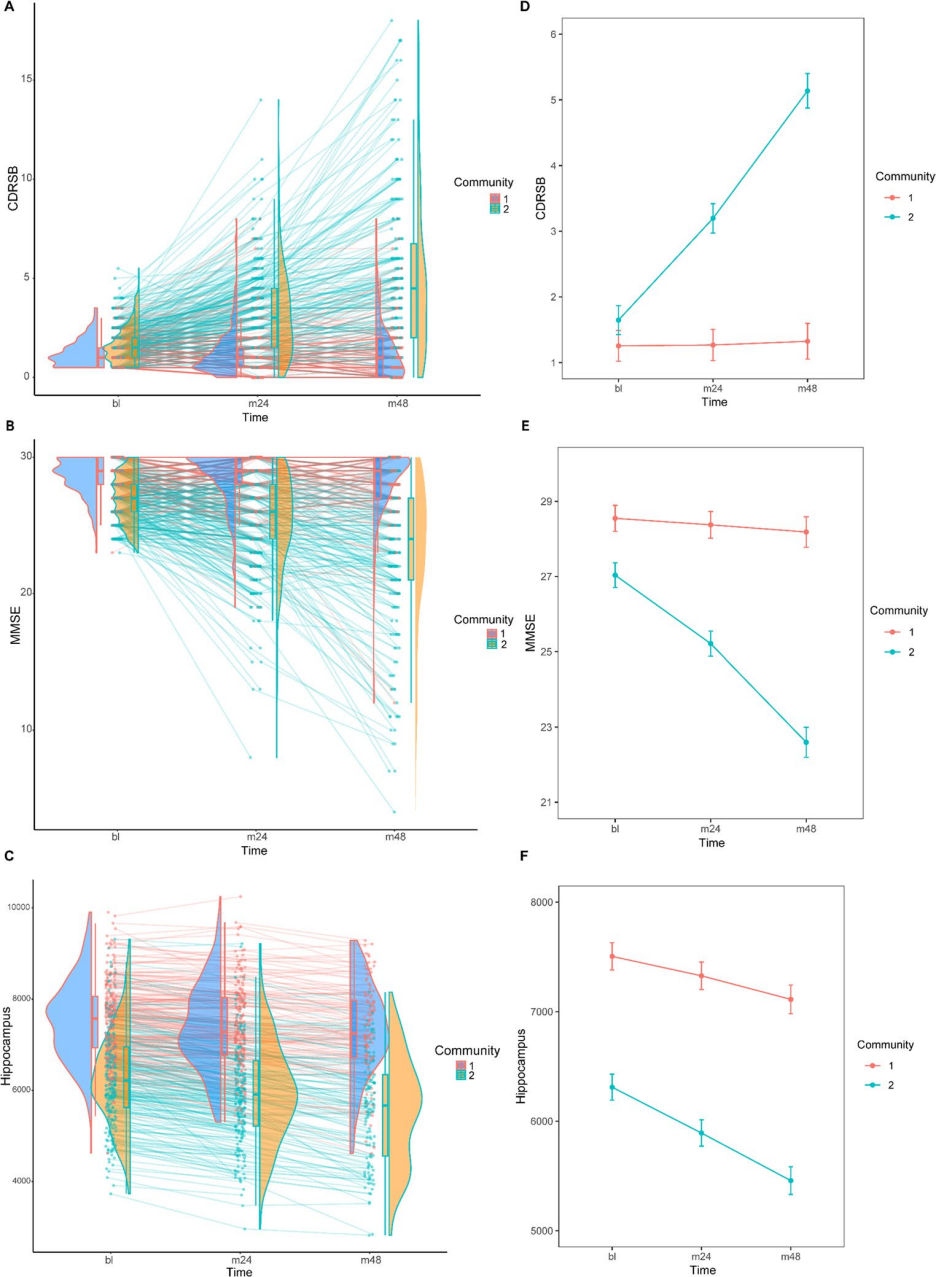
Longitudinal comparison of differences in different characteristics of MCI subgroups. (**A**–**C**) The plots depict longitudinal changes in cognitive scores and hippocampus atrophy, measured at baseline (bl), at the second-year follow-up (m24) and the fourth-year follow-up (m48). Longitudinal changes are shown for CDRSB scores (**A**), MMSE scores (**B**), and hippocampus atrophy (**C**). Longitudinal changes in estimated marginal means of CDRSB scores (**D**), MMSE scores (**E**), and hippocampus atrophy (**F**), displayed by the two MCI subgroups. Mean (± s.e.m.) scores for the different subtypes (MCI-C1, MCI-C2) during the baseline, the second-year follow-up (m24) and the fourth-year follow-up (m48)

Although our findings suggested significant differences in various characteristics between the MCI-C1 and MCI-C2 subgroups at baseline, this did not mean that the two subtypes remained significantly different over time. Therefore, we investigated whether the MCI-C1 and MCI-C2 subgroups still differed in various characteristics in the longitudinal data and in terms of progression to AD. Cognitive scores and brain characteristics demonstrated significantly different trends over time between the two MCI subgroups (Fig. 4A-C). We used linear mixed models to compare changes in cognitive and brain characteristics from baseline to the fourth year of follow-up, with characteristic scores as the dependent variable, communities (MCI-C1 and MCI-C2), time (baseline and follow-up), and the interaction between the two as fixed effects, and patient number as a random effect. Focusing on CDRSB scores (Fig. 4D), the results of the linear mixed model analysis revealed that both community ($F=~235.58$, $p<2.2 \times {10^{ - 16}}$) and time ($F=151.59$, $p<2.2 \times {10^{ - 16}}$) had significant effects on CDRSB scores, and that the interaction of these two (community*time;$F=140.02$, $p<2.2 \times {10^{ - 16}}$) also significantly affected CDRSB scores. Overall, over the time of the test, the MCI-C2 group’s CDRSB scores changed more noticeably. The CDRSB scores of the two MCI subtypes presented significant differences at baseline, the two-year follow-up period, and the four-year follow-up period (emmeans post hoc; bl: $p<{\mathrm{0}}{\mathrm{.0171}}$, m24: $p<0.{\mathrm{0001}}$, m48: $p<0.{\mathrm{0001}}$), and the CDRSB score of MCI-C2 was significantly higher than the score of MCI-C1. Similarly, in the analysis of MMSE scores (Fig. 4E), a significant interaction (community*time; $F=83.295$, $p<2.2 \times {10^{ - 16}}$) was observed, along with significant effects for community ($F=~312.556$, $p$<2.2$\times$10^-16^), and time ($F=115.740$, $p$<2.2$\times$10^-16^). At different time points, there were significant differences between the two MCI subtypes’ MMSE scores (emmeans post hoc; bl: $p<0.{\mathrm{0001}}$, m24: $p<0.{\mathrm{0001}}$, m48: $p<0.{\mathrm{0001}}$). The MMSE score of MCI-C2 was considerably lower than the score of MCI-C1. Likewise, a significant interaction ($F=55.841$, $p$<2.2$\times$10^-16^) as well as significant effects for time ($F=395.839$, $p$<2.2$\times$10^-16^) and community ($F=273.066$, $p$<2.2$\times$10^-16^) were noted for hippocampus atrophy (Fig. 4F). The hippocampus atrophy of the two MCI subtypes differed significantly (emmeans post hoc; bl: $p<0.{\mathrm{0001}}$, m24: $p<0.{\mathrm{0001}}$, m48: $p<0.{\mathrm{0001}}$), with MCI-C2 having a considerably higher levels of hippocampus atrophy than MCI-C1.

**Fig. 5:**
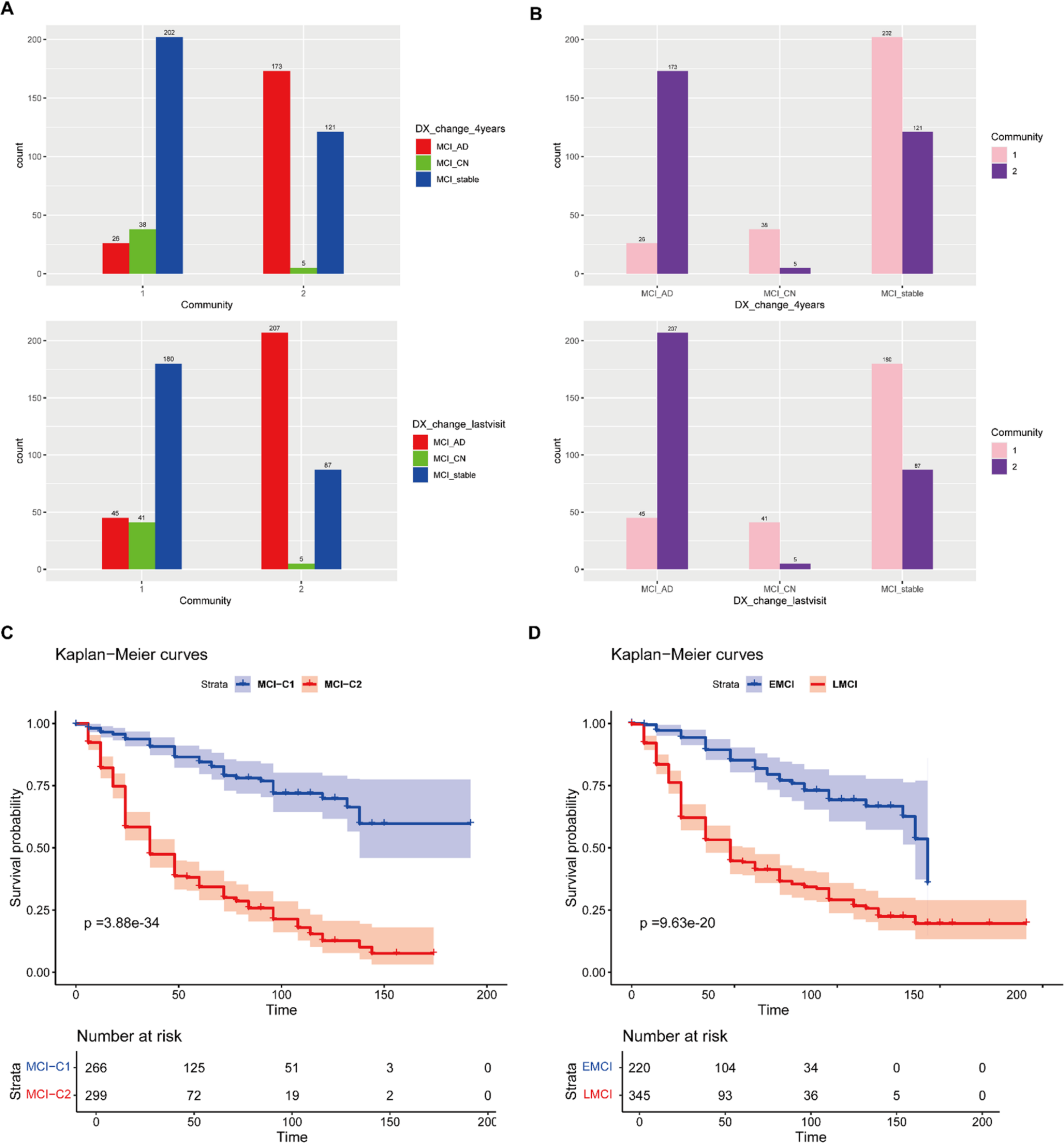
Longitudinal comparison of progression to AD in MCI subgroups. (**A**) Distribution of the three evolutionary groups (MCI_AD: MCI to AD; MCI_CN: MCI to CN; MCI_stable: stable MCI) in different communities based on diagnosis at the fourth and the last visit of follow-up. (**B)** Distribution of communities in three evolutionary groups. (**C**) Kaplan-Meier survival curve for MCI-C1 and MCI-C2 subtypes. (**D**) Kaplan-Meier survival curves for EMCI and LMCI subtypes

Furthermore, we assessed whether the MCI-C1 and MCI-C2 subgroups also showed differences in the progression of AD (Fig. 5). After four years, 173 patients (57.86%) in the MCI-C2 group became AD, five patients (1.67%) reversed to CN, and 121 patients (40.47%) remained MCI, but 26 patients (9.78%) in the MCI-C1 group became AD, 38 (14.29%) reversed to CN and 202 (75.94%) remained MCI. At the last follow-up, 207 (69.23%) in the MCI-C2 group became AD, five (1.67%) became CN, and 87 (29.10%) stayed MCI, while 45 (16.92%) in the MCI-C1 group became AD, 41 (15.41%) became CN, and 180 (67.67%) remained MCI. Moreover, the results of the survival analysis showed significant differences in AD progression between the two MCI groups ($p=3.88 \times {10^{ - 34}}$), with the MCI-C2 group progressing more rapidly compared to the MCI-C1 group (Fig. 5C). It is worth pointing out that the difference in survival was even more significant in our subgroups than in the early and late MCI subtypes (EMCI and LMCI) (Fig. 5D, $p=9.63 \times {10^{ - 20}}$).

Besides, a multivariate Cox proportional hazards regression was performed on the MCI cohort to assess the association between the identified subgroups (MCI-C1 and MCI-C2), demographic factors (age, education, sex), APOE genotype, and baseline cognitive scores (MMSE, CDRSB) with progression risk. The overall model was highly significant (likelihood ratio test = 256.5, *p* < 2.2 × 10⁻¹⁶). Subgroup membership, specifically MCI-C2 (HR = 4.16, *p* = 1.01 × 10⁻¹⁴), APOE4 = 2 (HR = 1.67, *p* = 0.0109), and higher baseline CDRSB scores (HR = 1.828, *p* < 2 × 10⁻¹⁶) were significantly associated with increased risk of progression, while higher MMSE scores were protective (HR = 0.907, *p* = 0.0120) (Fig. S6B). These findings confirm distinct progression risks between the two MCI subgroups.

### Validation of Molecular Subtypes by Trajectory Analysis

To validate the progression of severity between subtypes, we calculated clinical trajectories using the Elastic Principal Tree (EPT) algorithm by three types of data from all study subjects (Fig. S7). Firstly, the EPT was used to visualize the position in the tree for each patient category from healthy (CN) to MCI and AD (Fig. S7A-B). According to the findings, the patient’s risk category shifted as they move along the trajectory. Then, the healthy control group with the lowest probability of dementia was designated as the trajectory’s root node (Fig. S7C). Clinical trajectory regression analysis for trajectory 37–51 with pseudotime representing the degree of disease progression along the trajectory found that a high correlation (${r^2}=0.83$) between the classification of dementia subtypes and the pseudotime (Fig. S7D). These results validated the gradual change in dementia risk from CN-C1, MCI-C1, CN-C2, and MCI-C2 to AD, reflecting the trajectory of dementia.

## Discussion

In our study, we used similarity network fusion (SNF)-based framework to identify distinct AD subtypes by integrating three data modalities from all 972 subjects. Our study confirms the power of multimodal data integration for AD subtyping, as demonstrated by Avelar-Pereira et al. using multilayer networks (Avelar-Pereira et al. 2023). Unlike the longitudinal clustering model by Poulakis et al., which primarily relies on structural MRI to identify atrophy-based subtypes over time (Poulakis et al. 2022), our SNF-based method captures heterogeneous interactions across multiple data modalities simultaneously. Furthermore, while Dong et al.​ applied deep learning for AD/MCI classification based on sMRI (Dong et al. 2025), their emphasis was on accuracy rather than uncovering subtype-specific progression patterns, an approach that focuses on single-modality neuroimaging for classification tasks. Katabathula et al.​ included comorbidities in a clustering setup but used conventional mixed-data clustering (Katabathula et al. 2023), which may not handle heterogeneous data sources as effectively as network-based fusion. Young et al. introduced the machine-learning technique, the Subtype and Stage Inference (SuStaIn) model, to uncover data-driven disease phenotypes (Young et al. 2018), but it still relied heavily on structural MRI alone, without leveraging other information.

In our framwork, to optimize feature selection for the 14 initial cognitive measures, we excluded CDRSB and MMSE to avoid diagnostic circularity just as did by Pereira, B., et al. (Ning et al. 2021). To assess the impact of feature redundancy, we excluded four cognitive features (mPACCdigit_bl, mPACCtrailsB_bl, RAVLT_forgetting_bl, ADAS13_bl) based on correlation (Table S4) and group differences analysis (Table S5). Subtyping was repeated with the eight remaining features, yielding highly consistent clusters (ARI = 0.93) with the original 12-feature set, which was therefore retained for final analysis to preserve a comprehensive cognitive profile.

The seven MRI volumetric measures used here are established AD biomarkers, consistent with their use in recent predictive models of MCI conversion (Luo et al. 2024; Wu et al. 2024). In our data, these features also exhibited strong discriminative power for AD (Fig. S8), with AUCs ranging from 0.60 (ICV) to 0.77 (Hippocampus). This alignment with the literature supports their utility in profiling AD-related neuroanatomical changes. We employed the parameter set (*K* = 40, *α* = 0.5, *T* = 10) originally proposed for SNF (Jiao et al. 2024) to ensure reproducibility. To evaluate the sensitivity of the number of neighbors (*K*), we re-ran spectral clustering on the fused network using *K* values of 10, 20, 30, 40, and 50. High consistency (ARI > 0.8) between the *K* = 40 results and adjacent values confirmed robust clustering stability within a reasonable range around the chosen parameter.

Our subtyping analysis revealed a strong association between C2 and AD progression. The majority of CN and MCI individuals who later developed AD were assigned to C2—the same cluster that contained all baseline AD cases. Using only baseline multimodal data, we successfully distinguished progressive MCI (86.93% assigned to C2) from reversible MCI (88.37% assigned to C1). These results demonstrate the model’s ability to identify MCI cases at risk of AD progression versus those with a benign course, offering valuable insights for early intervention and patient stratification in AD, a topic of significant research interest (Luo et al. 2024; Edmonds et al. 2015; Lefort-Besnard et al. 2023).

The CN-C2 subgroup, identified by our method, exhibited more similar AD-like characteristics than CN-C1 across key biomarkers, including lower FDG-PET metabolism, higher ADAS-Cog 13 scores, and greater atrophy in the hippocampus and entorhinal cortex. GSVA revealed that the glycolysis and adipogenesis pathways were significantly upregulated in CN-C2 compared to CN-C1. Glycolysis, the metabolic pathway converting glucose to pyruvate for ATP production, is commonly dysregulated in neurodegenerative diseases such as Alzheimer’s and Parkinson’s (Bell et al. 2020; Tang 2020). There is growing evidence of the impact of adipose tissue endocrine activity on metabolic disorders and its relevance to AD risk (Bettinetti-Luque et al. 2024). Thus, abnormal glycolysis and adipogenesis processes may be early signals of AD and have the potential to develop into biomarkers for AD. Besides, our results suggested that CN-C2 was significantly up-regulated in the p53 pathway and MTORC1 signaling. Several studies have shown that individuals with AD had higher levels of p53 in several brain regions (Monte et al. 1997; Wolfrum et al. 2022). Interestingly, peripheral cells from patients with AD and MCI have been found to have a conformationally misfolded variant of p53, which has lost its typical structure and function (Abate et al. 2020). The presence of unfolded p53 then causes aberrant regulation, such as the promotion of the mTOR and CD44 pathways, which ultimately causes neuronal dysfunction and promotes neurodegeneration (Abate et al. 2020). mTOR signaling is involved in the pathogenesis of AD (Tramutola et al. 2017; Davoody et al. 2024). Therefore, p53 and mTOR signaling pathway appear to be potential drug targets for AD.

Because of the high risk of conversion of MCI to AD (10–15%)(Agostinho et al. 2024; Morris et al. 2001), an in-depth study of the classification of MCI is essential for early diagnosis and intervention of AD. Here, two MCI subtypes were obtained, which differed significantly in many key biomarkers. Significant differences in the prevalence of A/T/N biomarker abnormalities were observed between the MCI-C1 and MCI-C2 subgroups. Specifically, the MCI-C2 group exhibited a higher prevalence of abnormal CSF ABETA (A+), p-tau (T+), and FDG-PET (N+), constituting a more advanced pathological stage. This is consistent with the established clinical value of these biomarkers in predicting conversion from MCI to AD dementia(Mattsson et al. 2009; Gjerum et al. 2021; Caminiti et al. 2018). For instance, Caminiti et al. identified FDG-PET-SPM maps and CSF Aβ42 as top predictors of conversion (Caminiti et al. 2018). These findings suggested that our data-driven classification approach yielded physiologically and clinically unique subgroups of MCI.

Furthermore, from the longitudinal analyses, cognitive scores and hippocampal atrophy deteriorated more rapidly in the MCI-C2 subgroup than in MCI-C1. We observed that the CDRSB scores of the MCI-C2 subgroup were significantly higher than those of the MCI-C1 subgroup, and the MMSE scores of the MCI-C2 subgroup were significantly lower than those of the MCI-C1 subgroup. More importantly, the gap between the two groups on these scores increased with follow-up time. For example, the mean CDRSB score for MCI-C2 at the fourth-year follow-up time was 5.07, while it was only 1.29 for MCI-C1. Tzeng et al. examined the effect of baseline CDRSB on disease progression to AD or return to CN, and they found that the rate of conversion to AD increased with increasing CDRSB scores. In contrast, the rate of reversal to CN decreased with increasing CDRSB scores (Tzeng et al. 2022). This agreements with our findings that MCI-C2 had a higher proportion of patients converted to AD, and MCI-C1 had a higher proportion of patients converted to CN. MMSE is an essential marker of AD progression, with scores decreasing as the disease progresses (Honjo et al. 2024). In our study, in the fourth year of follow-up, the mean MMSE score for MCI-C2 was 22.76, implying mild AD (MMSE 21–26) (Henneges et al. 2016). Our results showed higher levels of MCI-C2 hippocampal atrophy, which may indicate a higher likelihood of conversion to AD in this group, as hippocampal atrophy is the initial stage in the development of AD, and many patients with hippocampal atrophy eventually evolve into AD (Josephs et al. 2017; Dhikav and Anand 2011; Xiao et al. 2023). There were notable differences in AV45 and FDG-PET between the two MCI subgroups. ABETA deposition assessed by AV45 is able to predict progression to AD in patients with MCI (Pfeil et al. 2021; Pascoal et al. 2020) and its abnormalities generally precede the onset of dementia (Jansen et al. 2015). Similarly, FDG-PET positivity was more prevalent in the MCI-C2 subgroup, a finding consistent with that FDG-PET-positive subjects were more likely to progress to AD clinically (Iaccarino et al. 2019).

In addition, GSVA revealed significant pathways and GO terms that differed significantly between MCI-C2 and MCI-C1. Most of the pathways upregulated in MCI-C2 were associated with AD. For example, GnRH was regarded as a critical pathogenic factor in AD (Nuruddin et al. 2014), and a clinical trial of a GnRH analog was currently being conducted as a possible treatment for AD (Wickramasuriya et al. 2022). Abnormalities in the VEGF signaling pathway were associated with AD, and targeting specific VEGFRs (VEGF receptors) may be a promising approach for treating AD (Ceci et al. 2024). The insulin signaling pathway plays a vital role in AD pathologies, and antidiabetic agents such as vanadium compounds can prevent or delay the onset of tau pathology by modulating insulin signaling pathways (Yao et al. 2023). As for BP terms, positive regulation of vascular associated smooth muscle cell (VSMC) differentiation and regulation of sphingolipid biosynthetic process were up-regulated in MCI-C2, both of which are important processes for AD. A study reported that dysfunctional VSMCs were involved in the pathogenesis of AD, and VSMCs may be a potential therapeutic target for AD (Aguilar-Pineda et al. 2021). Sphingolipids (SPLs) were reported closely associated with each pathogenesis (ABETA, tau, APOE, and α-Syn) of AD (Wang et al. 2024). Aberrant regulation of these crucial pathways and BP was observed in MCI-C2, suggesting that MCI-C2 developed to AD at a faster rate. And our survival analysis did demonstrate that MCI-C2 progressed to AD more quickly and trajectory analysis also revealed that MCI-C2 was closer to AD patients, which is consistent with these findings. Therefore, the clinical significance of the two MCI subtypes was reconfirmed by various analyses.

There were still several limitations in our study. First, although the ADNI consortium is a broadly collaborative program and has a comprehensive database of older patients, the majority of its participants remain white. Second, more detailed categorization is also needed in order to provide more rational treatment options for patients. Third, larger cohorts and validation in independent external datasets (e.g., NACC, AIBL) are essential to enhance the reliability and robustness of the findings.

This study presents a novel application of SNF to integrate cognitive, genetic, and structural MRI data, enabling the simultaneous identification of disease subtypes in AD. The key innovation lies in the unsupervised discovery of two biologically distinct MCI subtypes with differential risks of progression to AD, validated by longitudinal biomarker trajectories and differential pathway activities (such as, GnRH, VEGF signaling).

## Supplementary Information

Below is the link to the electronic supplementary material.

Supplementary Material 1 (DOCX 3.31 MB)

## Data Availability

The multi-omics data can be accessed from the Alzheimer’s Disease Neuroimaging Initiative (ADNI) database (http://adni.loni.usc.edu/) by applying for a data access permission.
