# Supplementary material for: Resolving Heterogeneity in the Diagnosis of Alzheimer’s Disease and its Progression Using Multimodal Data
Source: J Mol Neurosci. 2026 Feb 4;76(1):24. doi: 10.1007/s12031-026-02474-4 (PMC12872672; doi:10.1007/s12031-026-02474-4)
Supplement: Supplementary file 1 — Supplementary Material 1 (DOCX 3.31 MB) [file 12031_2026_2474_MOESM1_ESM.docx]

**Table S1** ADNI dataset descriptions coming from ADNIMERGE data set

| **Attribute** | **Description** | **Min. Value** | **Max. Value** | **Mean** |
| --- | --- | --- | --- | --- |
| **CDRSB** | Clinical Dementia Rating Scale Sum of Boxes | 0 | 7 | 1.02 |
| **ADAS11** | 11 item-AD Cognitive Scale (Score) | 0 | 37 | 8.7 |
| **ADAS13** | 13 item-AD Cognitive Scale (Score) | 1 | 52 | 13.87 |
| **ADASQ4** | 4 item-AD Cognitive Scale (Score) | 0 | 10 | 4.55 |
| **MMSE** | Mini-Mental State Examination | 20 | 30 | 28.09 |
| **RAVLT_immediate** | Rey’s Auditory Verbal Learning Test | 3 | 70 | 38.39 |
| **RAVLT_learning** | (scores for immediate response, learning, forgetting and percentage forgetting) | 2 | 12 | 4.78 |
| **RAVLT_forgetting** |  | 5 | 15 | 4.28 |
| **RAVLT_perc_forgetting** |  | −100 | 100 | 51.22 |
| **LDELTOTAL** | Logical memory delayed recall total score | 0 | 23 | 8.58 |
| **TRABSCOR** | Trail Making Test Part B Time | 32 | 300 | 101.64 |
| **FAQ** | Functional Activities Questionnaire | 0 | 22 | 2.28 |
| **mPACCdigit** | The modified preclinical Alzheimer cognitive composite using digit symbol substitution test | −21.23 | 6.3 | −3.96 |
| **mPACCtrailsB** | The modified preclinical Alzheimer cognitive composite using trail-making test part B | −21.23 | 5.44 | −3.69 |
| **Ventricles** | Volume of ventricles | 5650 | 145115 | 38019.3 |
| **Hippocampus** | Volume of hippocampus | 3731 | 10769 | 7028.3 |
| **WholeBrain** | Volume of Whole brain | 754507 | 1443990 | 1039992.53 |
| **Entorhinal** | Volume of the entorhinal cortex | 1558 | 5896 | 3651.27 |
| **Fusiform** | Volume of the fusiform gyrus | 10309 | 29950 | 17831.96 |
| **MidTemp** | Volume of middle temporal gyrus | 10234 | 29435 | 20053.78 |
| **ICV** | Intra Cranial Volume | 1129070 | 2072470 | 1537819.25 |
| **APOE4** | APOE4 gene presence | 0 | 2 | - |
| **PHS** | Polygenic hazard score | 1.24 | 2.82 | 0.29 |

**Table S2** Summary of results

| **SNF communities** | | **C1** | **C2** | **Total** |
| --- | --- | --- | --- | --- |
| Diagnosis at baseline | CN  % within diagnosis | 337  91.1% | 33  8.9% | 370  100% |
|  | MCI  % within diagnosis | 266  47.1% | 299  52.9% | 565  100% |
|  | AD  % within diagnosis | 0  0% | 37  100% | 37  100% |
| Diagnosis at the fourth year of follow-up | CN  % within diagnosis | 341  93.2% | 25  6.8% | 366  100% |
|  | MCI  % within diagnosis | 232  63.9% | 131  36.1% | 363  100% |
|  | AD  % within diagnosis | 30  12.3% | 213  87.7% | 243  100% |
| Diagnosis at last visit | CN  % within diagnosis | 300  94.9% | 16  5.1% | 316  100% |
|  | MCI  % within diagnosis | 235  70.4% | 99  29.6% | 334  100% |
|  | AD  % within diagnosis | 68  21.1% | 254  78.9% | 322  100% |

**Table S3** The prevalence of biomarker profiles for MCI-C1 and MCI-C2

|  | **A-T-(N)-** | **A-T-(N)+** | **A-T+(N)-** | **A-T+(N)+** | **A+T-(N)-** | **A+T-(N)+** | **A+T+(N)-** | **A+T+(N)+** |
| --- | --- | --- | --- | --- | --- | --- | --- | --- |
| **MCI-C1** | 81 (30.5%) | 11  (4.1%) | 41  (15.4%) | 19  (7.1%) | 23  (8.6%) | 10  (3.8%) | 58  (21.8%) | 23  (8.6%) |
| **MCI-C2** | 16 (5.4%) | 6  (2.0%) | 15  (5.0%) | 10  (3.3%) | 14 (  4.7%) | 20 (6.7%) | 98 (32.8%) | 120  (40.1%) |

**Table S4** The correlation between 12 cognition features

|  | ADAS11_bl | ADAS13_bl | ADASQ4_bl | RAVLT_immediate_bl | RAVLT_learning_bl | RAVLT_forgetting_bl | RAVLT_perc_forgetting_bl | LDELTOTAL_BL | TRABSCOR_bl | FAQ_bl | mPACCdigit_bl | mPACCtrailsB_bl |
| --- | --- | --- | --- | --- | --- | --- | --- | --- | --- | --- | --- | --- |
| ADAS11_bl | 1 | **0.96** | 0.72 | -0.68 | -0.54 | 0.18 | 0.53 | -0.63 | 0.47 | 0.51 | -0.77 | -0.77 |
| ADAS13_bl | **0.96** | 1 | 0.87 | -0.73 | -0.59 | 0.22 | 0.6 | -0.68 | 0.5 | 0.54 | **-0.85** | **-0.85** |
| ADASQ4_bl | 0.72 | 0.87 | 1 | -0.69 | -0.58 | 0.29 | 0.61 | -0.65 | 0.36 | 0.46 | **-0.84** | **-0.82** |
| RAVLT_immediate_bl | -0.68 | -0.73 | -0.69 | 1 | 0.62 | -0.24 | -0.64 | 0.6 | -0.42 | -0.42 | 0.69 | 0.71 |
| RAVLT_learning_bl | -0.54 | -0.59 | -0.58 | 0.62 | 1 | -0.03 | -0.46 | 0.5 | -0.29 | -0.37 | 0.57 | 0.57 |
| RAVLT_forgetting_bl | 0.18 | 0.22 | 0.29 | -0.24 | -0.03 | 1 | 0.8 | -0.21 | 0.01 | 0.1 | -0.22 | -0.2 |
| RAVLT_perc_forgetting_bl | 0.53 | 0.6 | 0.61 | -0.64 | -0.46 | **0.8** | 1 | -0.52 | 0.24 | 0.38 | -0.59 | -0.57 |
| LDELTOTAL_BL | -0.63 | -0.68 | -0.65 | 0.6 | 0.5 | -0.21 | -0.52 | 1 | -0.38 | -0.49 | **0.83** | **0.84** |
| TRABSCOR_bl | 0.47 | 0.5 | 0.36 | -0.42 | -0.29 | 0.01 | 0.24 | -0.38 | 1 | 0.35 | -0.5 | -0.63 |
| FAQ_bl | 0.51 | 0.54 | 0.46 | -0.42 | -0.37 | 0.1 | 0.38 | -0.49 | 0.35 | 1 | -0.56 | -0.57 |
| mPACCdigit_bl | -0.77 | **-0.85** | **-0.84** | 0.69 | 0.57 | -0.22 | -0.59 | **0.83** | -0.5 | -0.56 | 1 | **0.97** |
| mPACCtrailsB_bl | -0.77 | **-0.85** | **-0.82** | 0.71 | 0.57 | -0.2 | -0.57 | **0.84** | -0.63 | -0.57 | **0.97** | 1 |

**Table S5** The pvalue of 12 cognition features by comparing CN and AD

| **Cognition features** | pvalue |
| --- | --- |
| ADAS11_bl | 1.17E-16 |
| ADAS13_bl | 3.18E-20 |
| ADASQ4_bl | 4.17E-35 |
| RAVLT_immediate_bl | 1.44E-22 |
| RAVLT_learning_bl | 3.60E-18 |
| RAVLT_forgetting_bl | 0.062540785 |
| RAVLT_perc_forgetting_bl | 5.14E-33 |
| LDELTOTAL_BL | 1.44E-66 |
| TRABSCOR_bl | 4.26E-10 |
| FAQ_bl | 1.77E-13 |
| mPACCdigit_bl | 1.36E-31 |
| mPACCtrailsB_bl | 3.29E-31 |


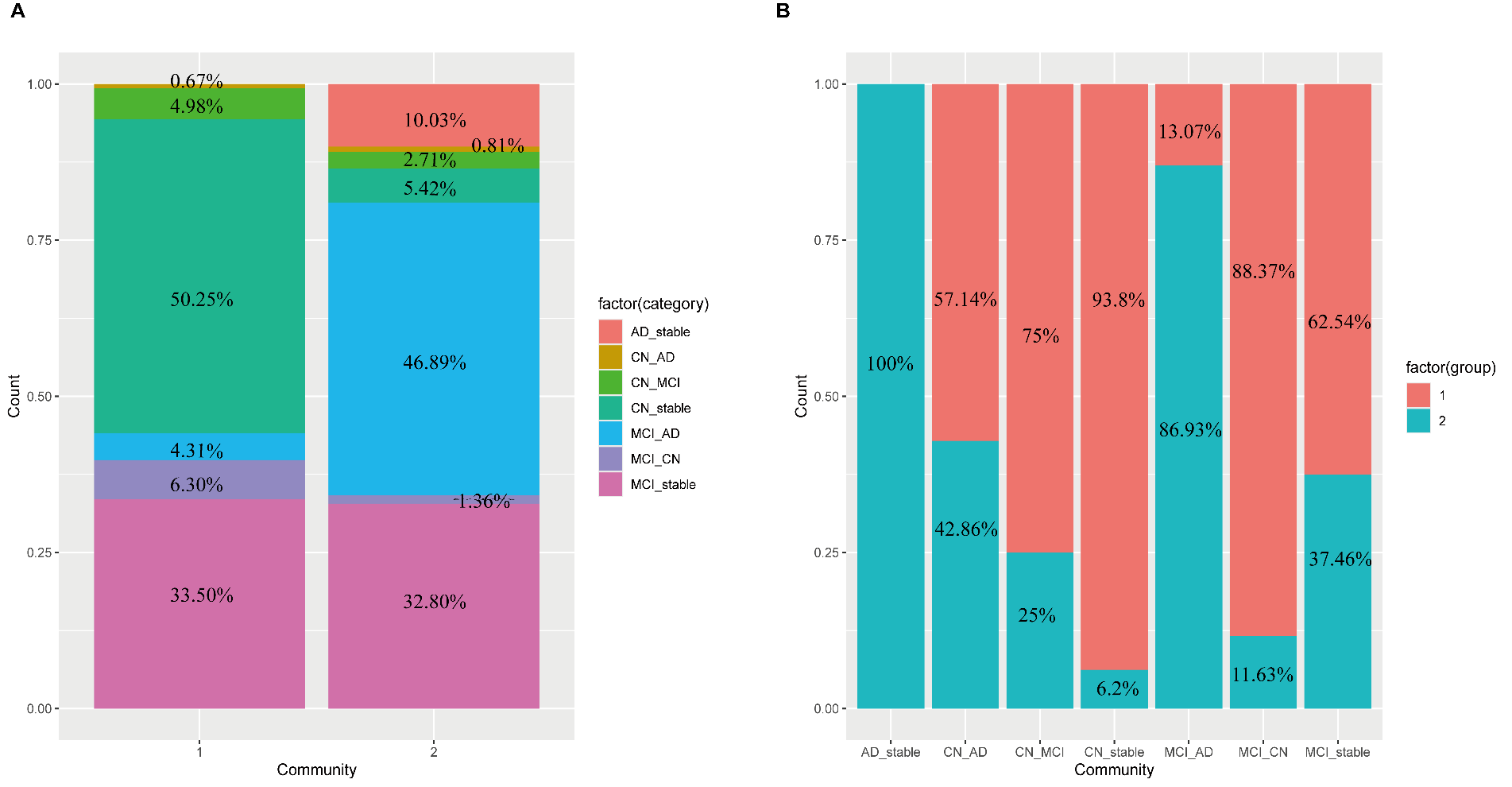


**Fig. S1 Longitudinal analysis of two** c**ommunities. A.** Percentages represent the proportion of each disease state in each community. **B**. Percentages represent the proportion of individuals in each category that belong to C1 or C2.


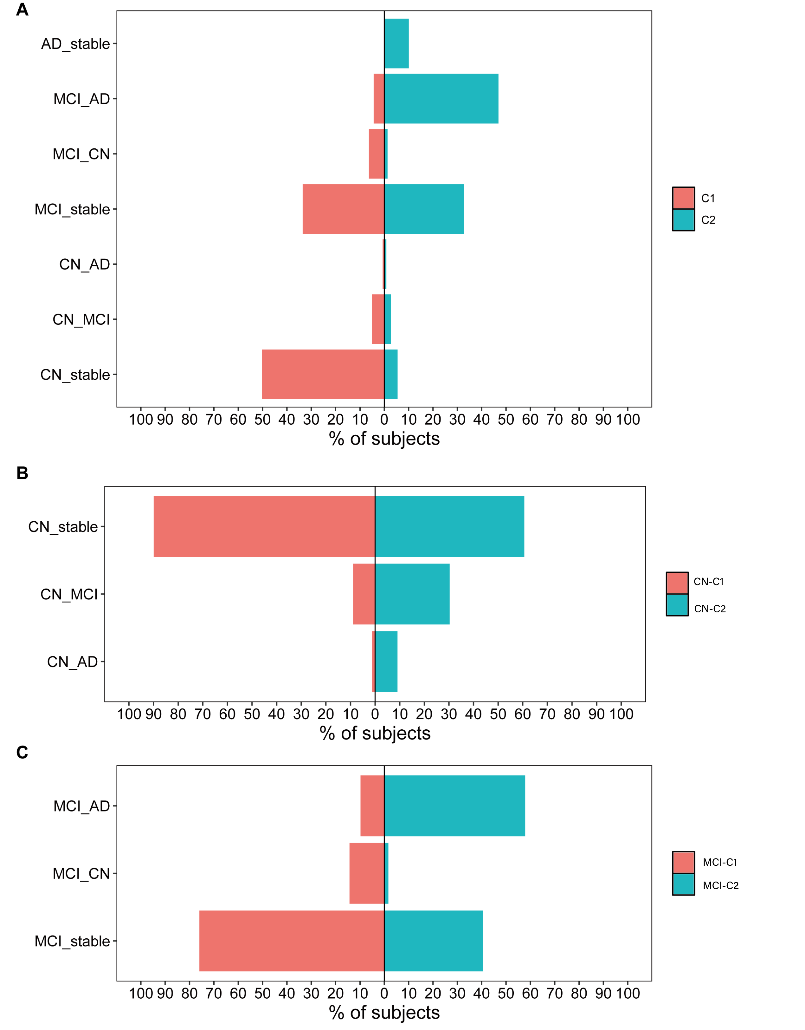


**Fig. S2** **Longitudinal analysis of two communities based on the diagnosis at the fourth year of follow-up. A.** Bidirectional bar shows the proportion of each of the disease states in C1 and C2, respectively. **B**. Bidirectional bar shows the proportion of each of the disease states in CN-C1 and CN-C2, respectively. **C**. Bidirectional bar shows the proportion of each of the disease states in MCI-C1 and MCI-C2, respectively.


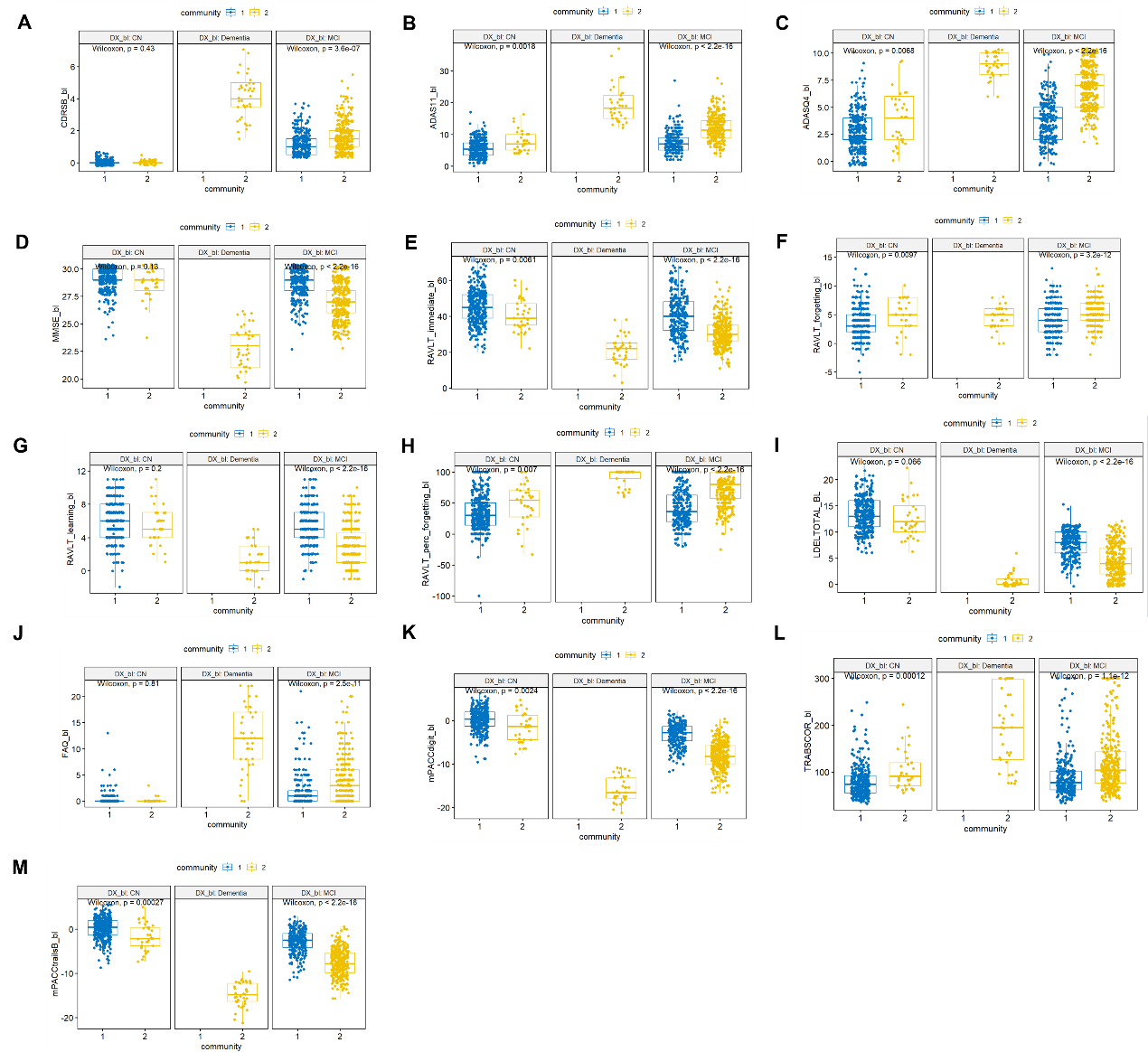


**Fig. S3 Comparing cognitive scores for CN, MCI and AD across different communities at baseline.** A. CDRSB; B. ADAS11; C. ADASQ4; D. MMSE; E. RAVLT_immediate; F. RAVLT_forgetting; G. RAVLT_learning; H. RAVLT_perc_forgetting; I. LDELTOTAL; J. FAQ; K. mPACCdigit; L. TRABSCOR; M. mPACCtrailsB.


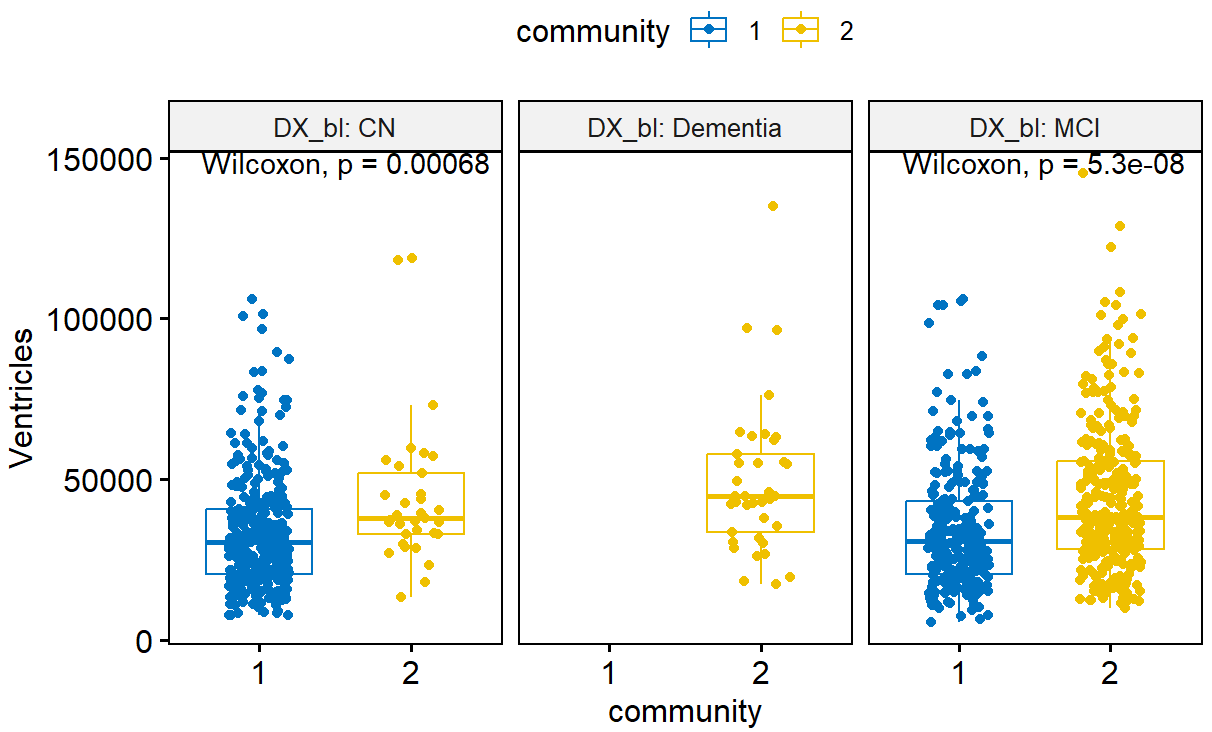

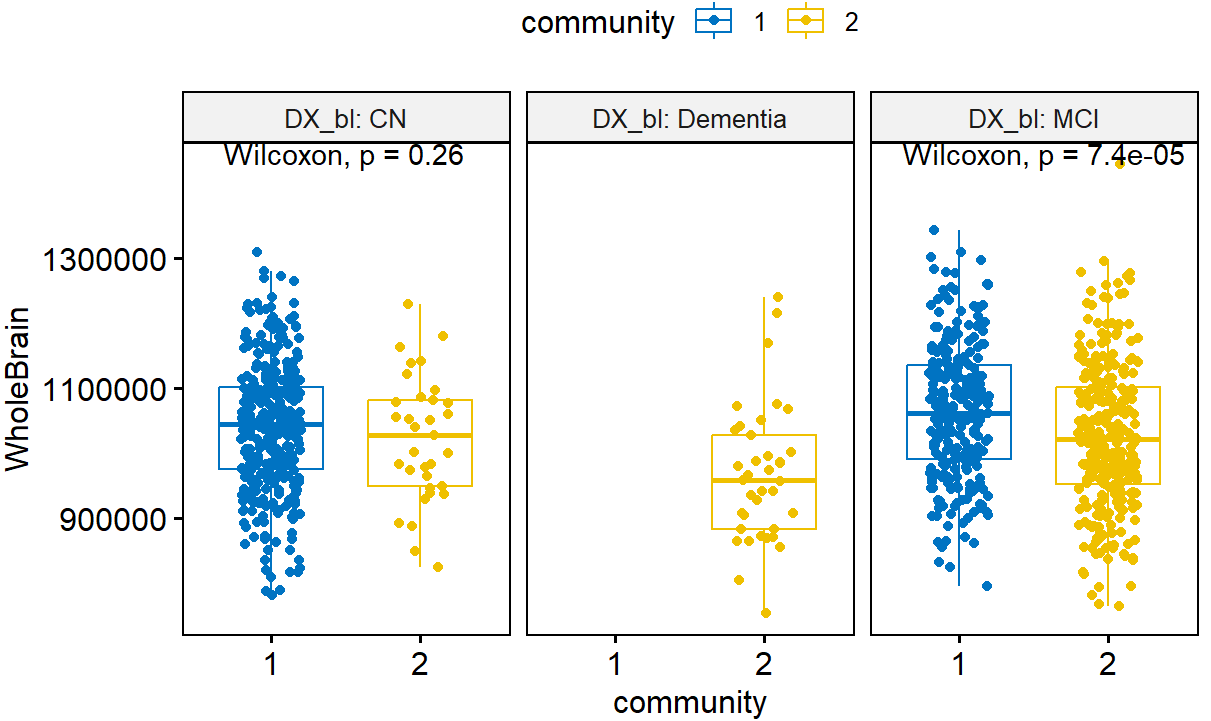

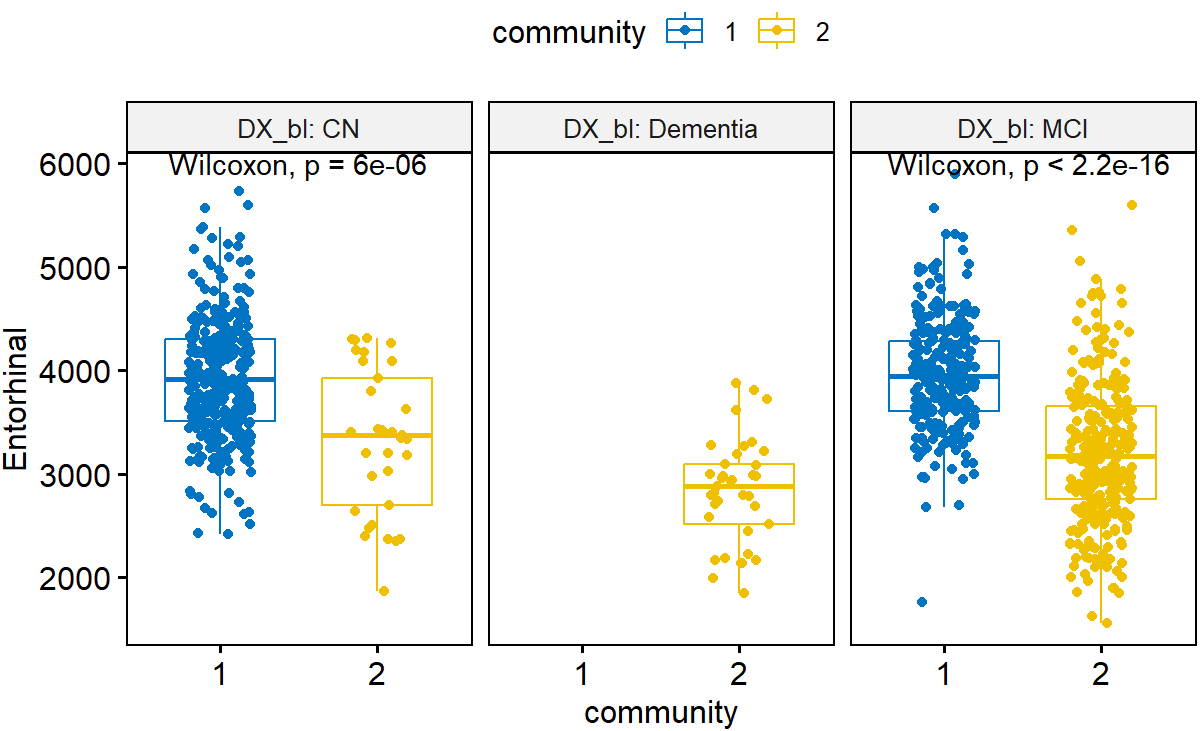

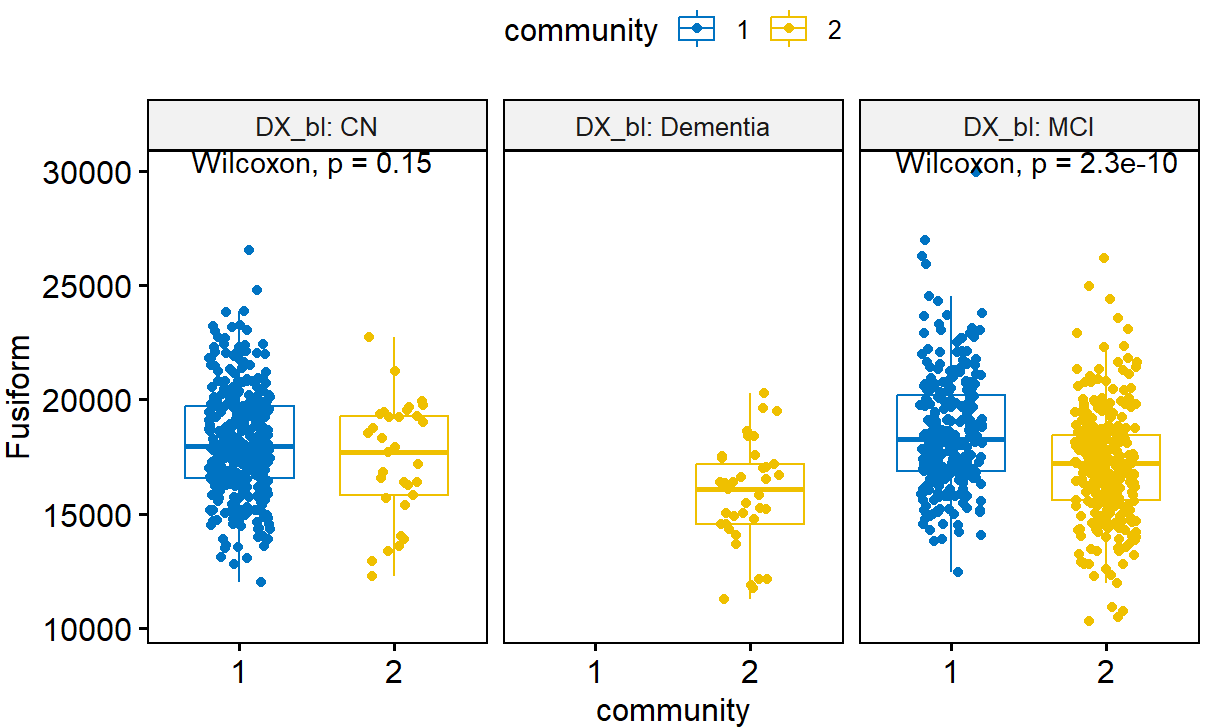

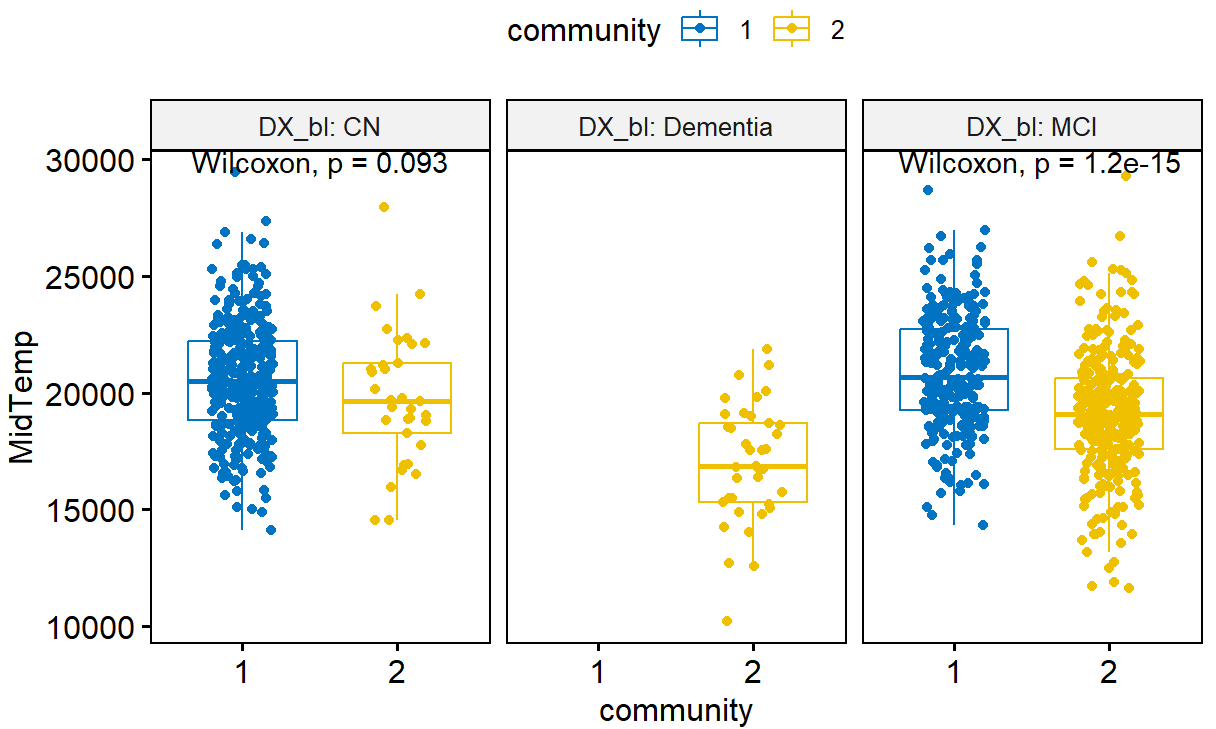

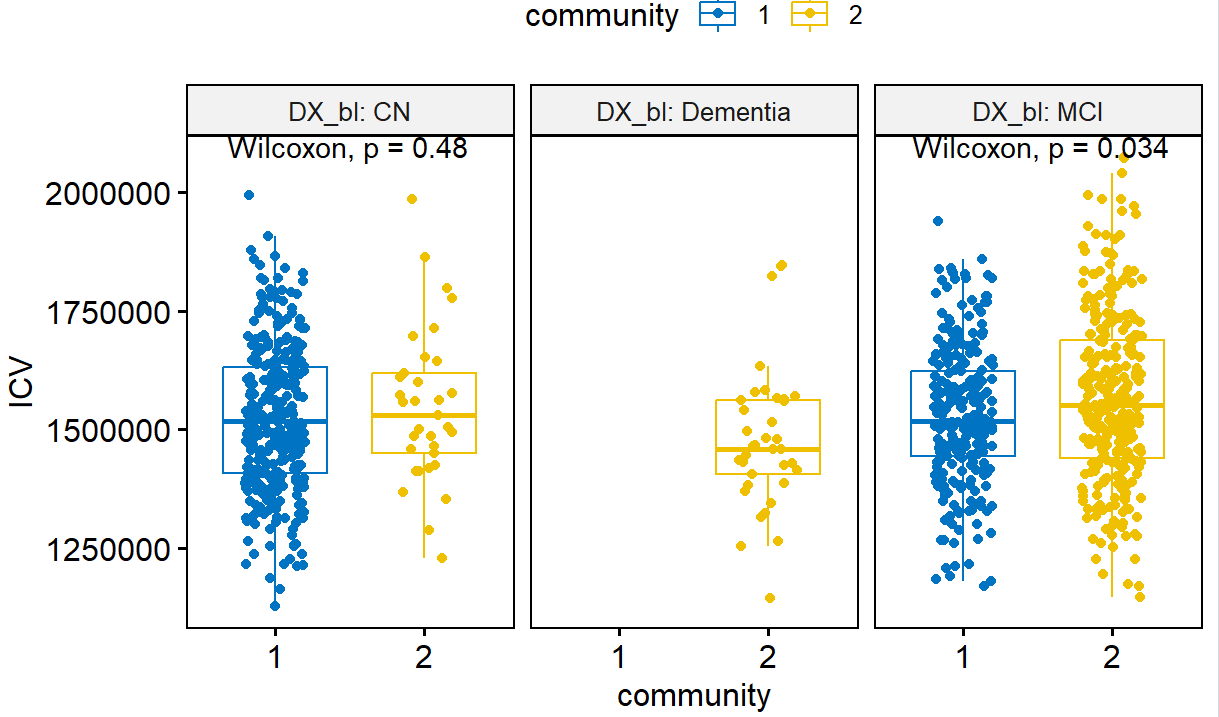


**A**

**C**

**F**

**E**

**D**

**B**

**Fig. S4 Comparing brain imaging data information for CN, MCI and AD across different communities at baseline.** A. Ventricles; B. WholeBrain; C. MidTemp; D. Entorhinal; E. Fusiform; F. ICV.

**
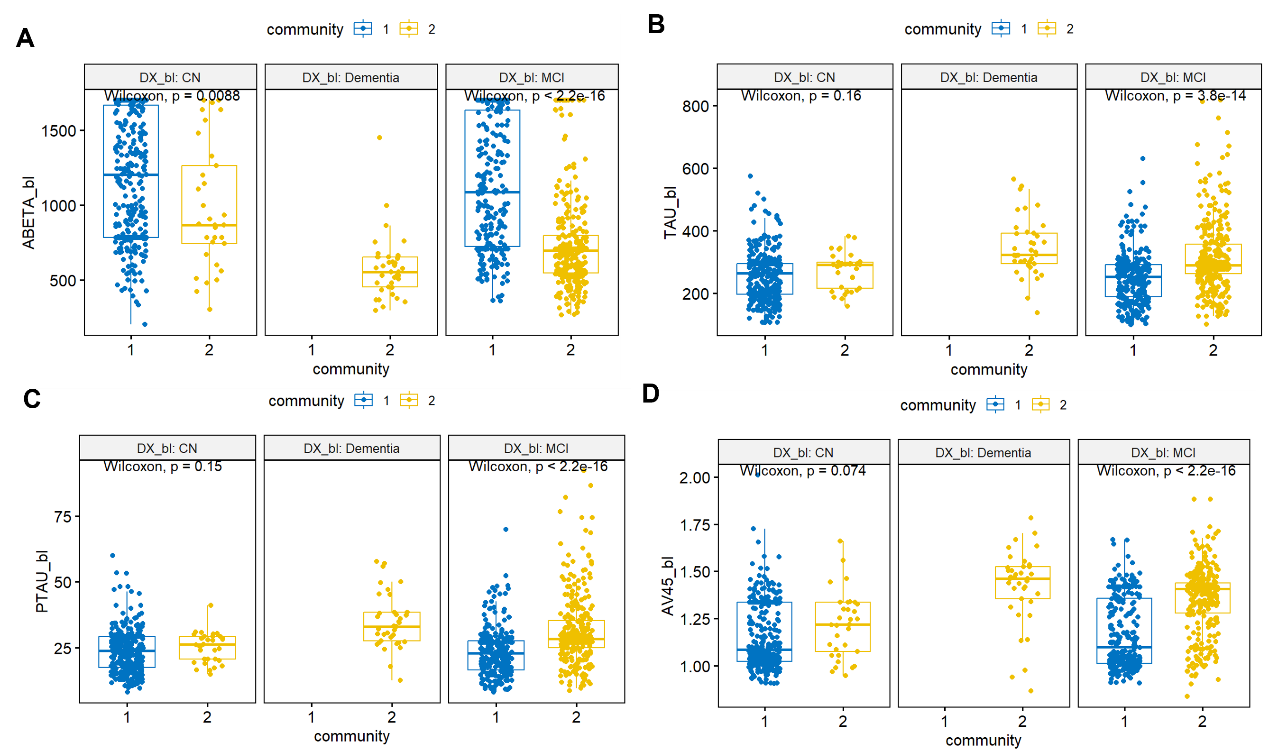
**

**Fig. S5 Comparing of CN, MCI and AD across different communities at baseline in** (A) ABETA; (B) TAU; (C) pTAU; (D) AV45.

**Fig. S6 Comparing MCI-C1 and MCI-C2.** (A)The top 20 up- and down-regulated GO BP terms that are significantly different between MCI-C2 and MCI-C1 revealed by GSVA. The p-value of each GO term is indicated adjacent to the bar. (B) Forest plot for Cox proportional hazards model.


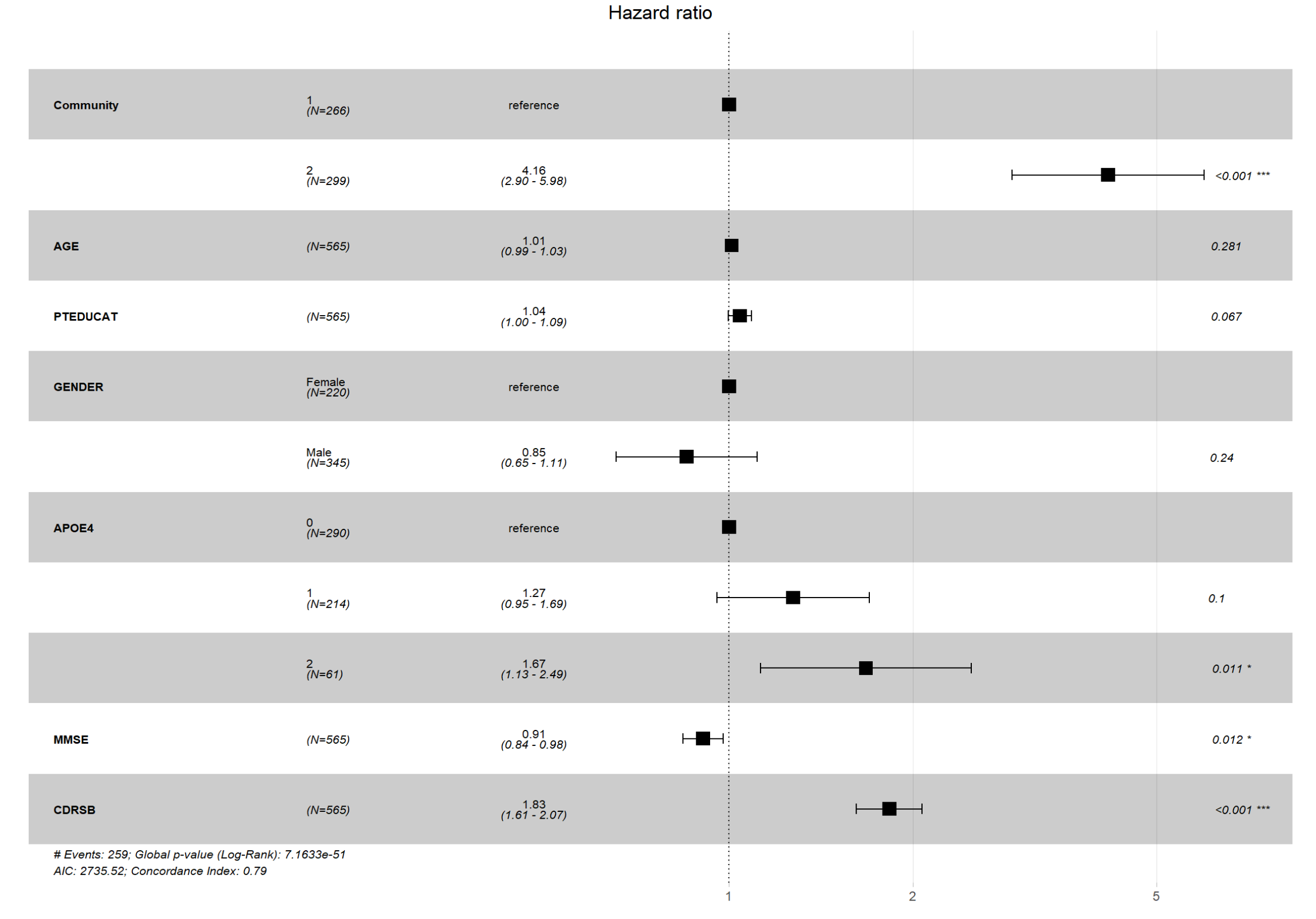


**A**


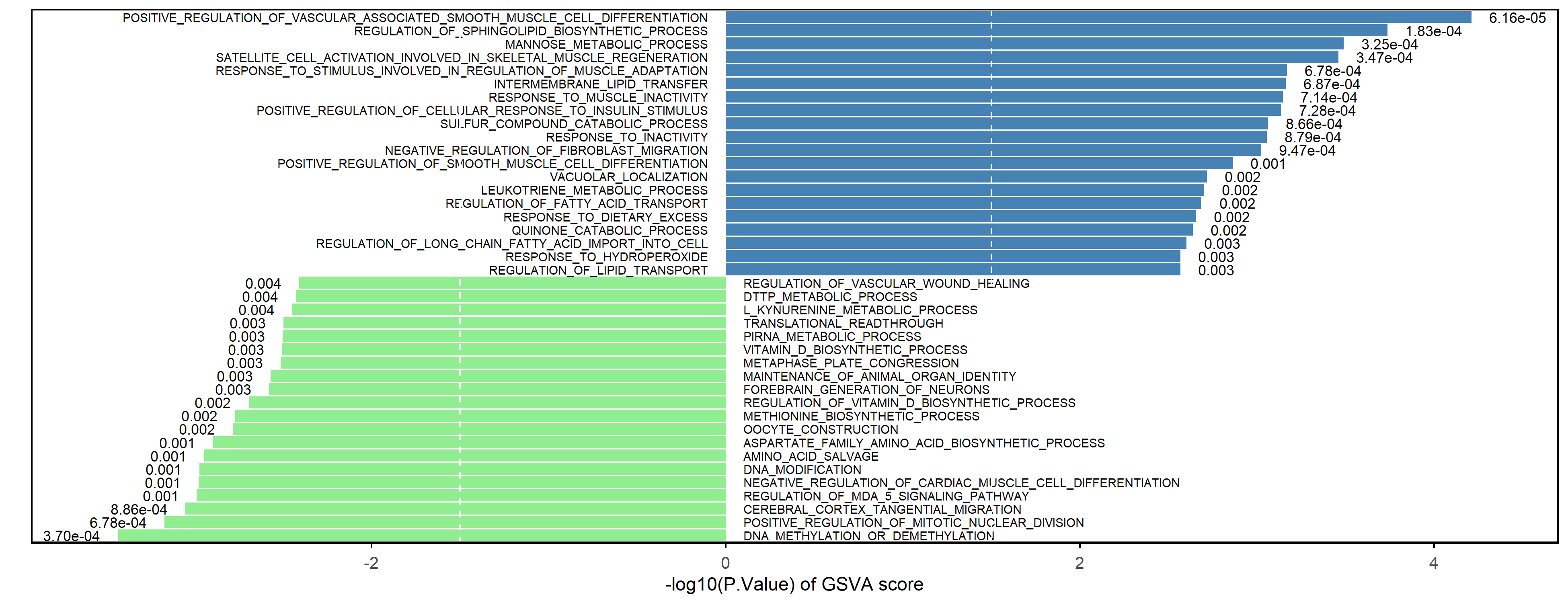


**B**


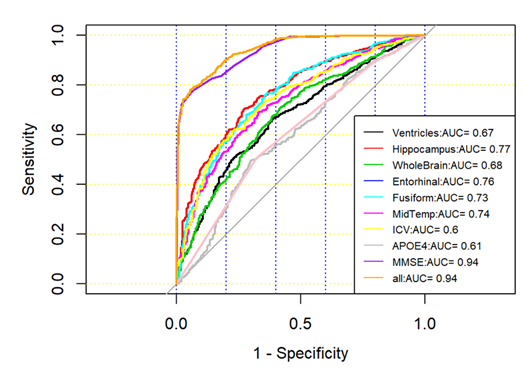
**
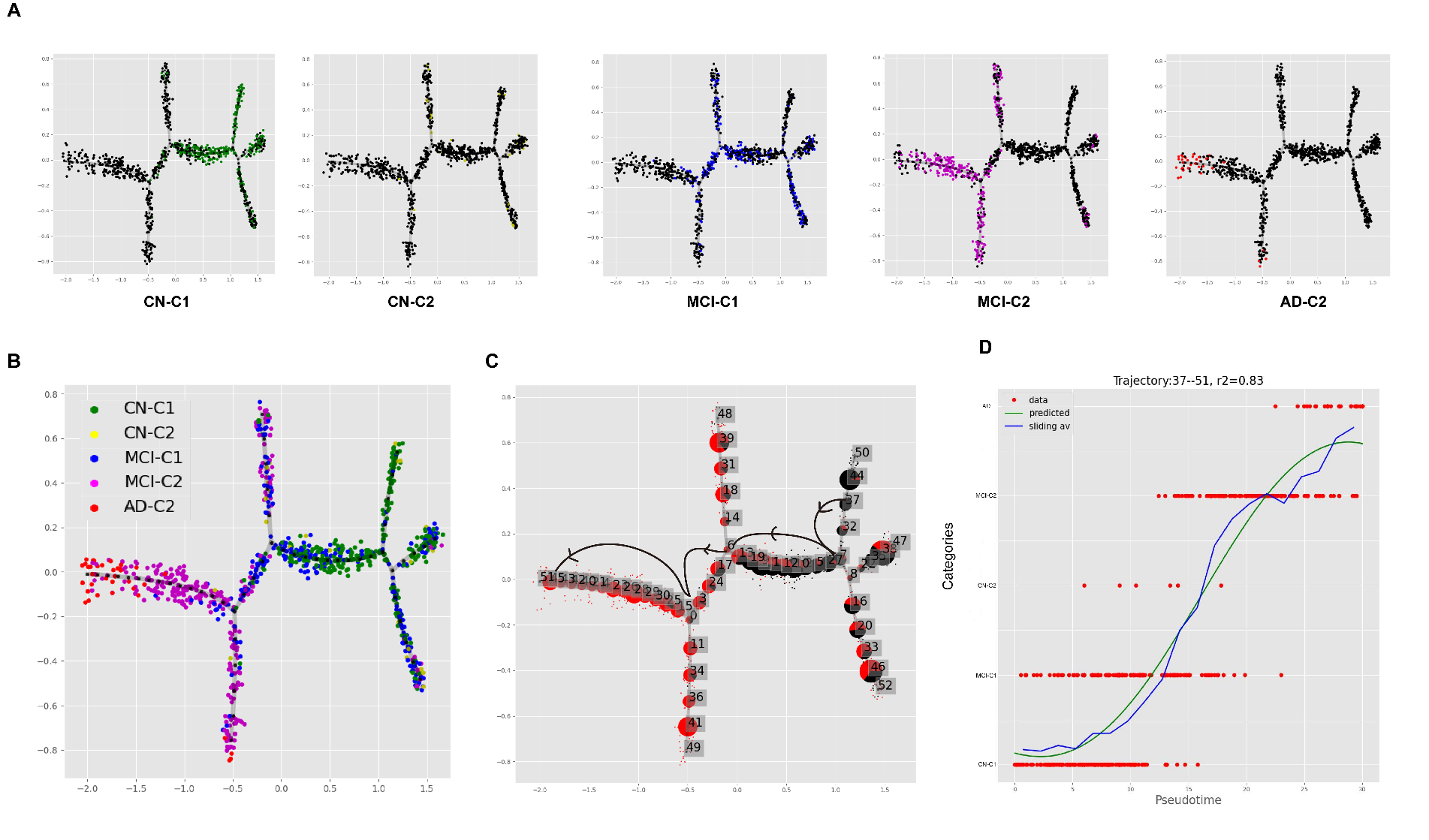
Fig. S7 Principal tree recapitulating the multidimensional structure of the Alzheimer's dataset.** A. On the trajectory tree, patient phenotypes were sorted from CN-C1, CN-C2 to MCI-C1, MCI-C2, and AD-C2 (dementia). B. The trajectory tree was colored differently to represent each patient phenotype. C. Normal controls were shown as root nodes, and patients at the highest risk of dementia were shown as final states. D. Regression analysis of clinical trajectories for trajectories 37-51, where pseudotime indicated the extent of disease progression along the trajectory.

**Fig. S8 Comparison of the Predictive Performance for Alzheimer’s Disease Using Different Feature Modalities by ROC Analysis**.
